# Efavirenz metabolism and CNS toxicity in Ugandan children: impact of CYP2B6 genotype and plasma metabolite profiles

**DOI:** 10.3389/fphar.2026.1778383

**Published:** 2026-04-24

**Authors:** Sandra Soeria-Atmadja, Madeleine Pettersson Bergstrand, Pauline Amuge, Sarah Nanzigu, Dickson Bbuye, Johanna Rubin, Adeodata Kekitiinwa, Celestino Obua, Marja-Liisa Dahl, Anton Pohanka, Lars L. Gustafsson, Lars Navér, Jaran Eriksen

**Affiliations:** 1 Department of Clinical Science, Intervention and Technology, Division of Pediatrics, Karolinska Institutet, Stockholm, Sweden; 2 Department of Pediatrics, Karolinska University Hospital, Stockholm, Sweden; 3 Division of Clinical Pharmacology, Department of Laboratory Medicine, Karolinska Institutet, Stockholm, Sweden; 4 Department of Clinical Pharmacology, Karolinska University Hospital, Stockholm, Sweden; 5 Baylor College of Medicine, Children’s Foundation-Uganda, Kampala, Uganda; 6 Kampala Institute of Science and Technology, Kyengera, Uganda; 7 Department of Clinical Pharmacology and Therapeutics, Makerere University, Kampala, Uganda; 8 Department of Research and Development, Alpha Center for Research Development, Mbarara, Uganda; 9 Unit of infectious diseases/Venhälsan, Södersjukhuset, Stockholm, Sweden; 10 Department of Global Public Health, Karolinska Institutet, Stockholm, Sweden; 11 Department of Clinical Science and Education, Karolinska Institutet, Stockholm, Sweden

**Keywords:** 7-hydroxyefavirenz, 8-hydroxy-efavirenz, adverse events, children, efavirenz, HIV, metabolites, Uganda

## Abstract

**Introduction:**

The non-nucleoside reverse transcriptase inhibitor efavirenz (EFV) is used in antiretroviral therapy (ART) against HIV. EFV is mainly metabolized by CYP2B6 to 8-hydroxyefavirenz (8-OH-EFV) and to a lesser extent by CYP2A6 to 7-hydroxyefavirenz (7-OH-EFV). Previous studies have only examined these metabolites in adults, indicating that EFV-hydroxy-metabolites might contribute to central nervous system (CNS) toxicity.

**Aims:**

This study aimed to quantify EFV and its metabolites including a recently identified EFV metabolite named EFAdeg in plasma and to explore their association with CYP2B6 metabolizer phenotypes and CNS adverse effects, in Ugandan children. Additionally, we examined signs of EFV autoinduction.

**Methods:**

We prospectively followed 99 Ugandan ART-naïve children in 2015–2016, aged 3–12 years with plasma sampling at 2, 6, 12, and 24 weeks after initiating-EFV-based ART. Using liquid chromatography high-resolution tandem mass spectrometry, we quantified EFV, 8-OH-EFV, 7-OH-EFV and EFAdeg in both unconjugated and conjugated forms. Genotyping for single nucleotide polymorphisms (SNP) in CYP2B6 and CYP2A6 was performed. CYP2B6 metabolizer phenotypes were predicted by the composite genotype of the two SNPs CYP2B6 c.516G>T and CYP2B6 c.983T>C. CNS adverse effects were assessed *via* a questionnaire. Autoinduction was investigated in a multivariate restricted maximum likelihood regression model (REML) with log_(e)_ ((8-OH-EFV + EFAdeg)/EFV) as the outcome variable.

**Results:**

Metabolite/EFV ratios in plasma varied significantly with CYP2B6 metabolizer phenotypes. Conjugated 8-OH-EFV (8-OH-EFV -glucuronide and 8-OH-EFV-sulfate) and conjugated EFAdeg constituted the major circulating forms of EFV in extensive and intermediate metabolizers, while the parent drug EFV dominated in slow metabolizers, who also displayed the highest levels of 7-OH-EFV. CNS symptoms were common, transient and mild and significantly associated with EFV plasma concentrations above 4,000 ng/mL and slow CYP2B6 metabolizer phenotype. None of the metabolites in plasma were associated with an increased risk of CNS toxicity. Evidence of autoinduction of EFV metabolism was observed among extensive metabolizers.

**Conclusion:**

This first study of EFV metabolites in children revealed distinct distribution patterns influenced by CYP2B6 metabolizer phenotype. Mild CNS-related adverse effects were associated with high EFV levels and slow CYP2B6 metabolizer phenotype, but not with EFV hydroxy metabolites. Autoinduction of EFV metabolism was observed in CYP2B6 extensive metabolizers.

## Introduction

Efavirenz (EFV) is a non-nucleoside reverse transcriptase inhibitor used to treat human immunodeficiency virus (HIV). Although dolutegravir (DTG)-based regimens are now the preferred first-line antiretroviral treatment (ART) for children, EFV combined with two nucleoside reverse transcriptase inhibitors (NRTIs) remains a WHO-recommended alternative for children over 3 years (World Health Organization) and continues to be used in settings where the transition to DTG has not been fully implemented due to programmatic and supply challenges (Desmonde et al., 2025).

EFV is well absorbed in healthy volunteers with peak plasma concentrations approximately 5 h after oral intake (Agency, 2009). EFV is highly albumin bound (>99%) (Avery et al., 2013a) with a plasma terminal half-life ranging from 35 to 115 h (Agency, 2009; Taylor et al., 2012) making once-daily dosing feasible.

Studies in adults show that EFV predominantly undergoes liver metabolism through hydroxylation to 8-hydroxyefavirenz (8-OH-EFV) by CYP2B6, with a minor CYP2A6 contribution (Ward et al., 2003; Ogburn et al., 2010). *In vitro* studies suggest that CYP3A4, CYP3A5, and CYP1A2 also contribute to the formation of 8-OH-EFV, albeit to a lesser extent (Ward et al., 2003). Around 80% of EFV is metabolized to 8-OH-EFV. The remainder is mainly hydroxylated to 7-hydroxyefavirenz (7-OH-EFV), by CYP2A6 and possibly by CYP2B6 (Ogburn et al., 2010). A small fraction undergoes direct glucuronidation to EFV-N-glucuronide (EFV-N-gln) (Cho et al., 2011; Belanger et al., 2009), Figure 1.

**FIGURE 1:**
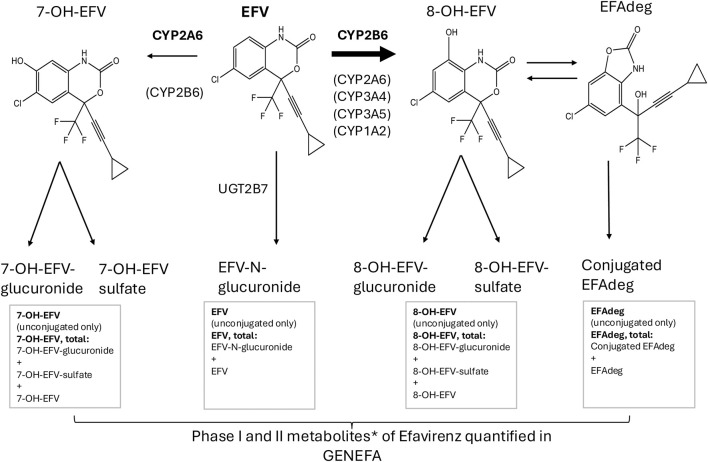
Efavirenz (EFV) and metabolites detected and quantified in GENEFA. Ninety-nine ART-naive Ugandan children aged 3–12 years were enrolled in GENEFA and initiated EFV-based antiretroviral therapy. Plasma EFV and metabolites were detected and quantified with a method using liquid chromatography, high-resolution tandem mass spectrometry. Total concentrations include both phase I (8-OH-EFV, 7-OH-EFV) and phase II metabolites as the phase II metabolites 8-OH-EFV glucuronide, 8-OH-EFV sulfate, 7-OH-EFV glucuronide, 7-OH-EFV sulfate and EFV-N-glucuronide were detected but could not be individually quantified. During method development a new substance was found, hypothesized to be an 8-OH-EFV degradation product (“EFAdeg”t). *Desta Z et al. Clinical Pharmacogenetics Implementation Consortium (CPIC) Guideline for CYP2B6 and Efavirenz-Containing Antiretroviral Therapy. Clin Pharmacol Ther. 2019 October; 106(4):726–733. doi: 10.1002/cpt.1477. Epub 2019 July 5. PMID: 31006110; PMCID: PMC6739160.

8-OH-EFV is further metabolized by CYP2B6 to 8,14-dihydroxy-EFV (6). The three hydroxylated phase I EFV-metabolites (8-OH-, 7-OH-, and 8,14-dihydroxy EFV), are either conjugated by multiple UDP-glucuronosyltransferase (UGT) isoforms or sulfated before renal excretion (Aouri et al., 2016; Mutlib et al., 1999). A study in adults found that the phase II metabolites 8-OH-EFV glucuronide and 8-OH -EFV sulfate were the major circulating metabolites with 64- and 7-fold higher concentrations than the unconjugated 8-OH-EFV (Aouri et al., 2016).

To date, no studies have described the metabolic pathways of EFV in children, even though it is known that developmental changes in both phase I enzymes such as cytochrome P450 and phase II enzymes responsible for conjugation may result in age-dependent variations in drug biotransformation (Croom et al., 2009; Kearns et al., 2003). A South African study found that children have lower (dose-adjusted) EFV plasma levels than adults due to higher clearance relative to their size (Sinxadi et al., 2015). Analysis of CYP2B6 protein from fetal and pediatric liver microsomal samples showed that CYP2B6 expression increases with age (Croom et al., 2009). Consequently, the distribution of EFV plasma metabolites may differ between children and adults. Furthermore, genetic variability in the main (CYP2B6) and secondary (CYP2A6, CYP3A5, and UGT2B7) pathways for EFV clearance could contribute to interindividual differences in metabolism (di Iulio et al., 2009; Kwara et al., 2009; Colic et al., 2015; Chala et al., 2023; Reay et al., 2017; Soeria-Atmadja et al., 2017; Viljoen et al., 2010; Holzinger et al., 2012; Mukonzo et al., 2014).

CNS-symptoms such as sleep disturbances, headaches, dizziness and neurocognitive difficulties are frequently reported in adults and children treated with EFV-based ART (Soeria-Atmadja et al., 2017; Marzolini et al., 2001; Arnab et al., 2023; Ford et al., 2015; Van de Wijer et al., 2017; Fumaz et al., 2005; Mukonzo et al., 2013; Van de Wijer et al., 2019). These may be non-severe (Soeria-Atmadja et al., 2017; Fumaz et al., 2005; Mukonzo et al., 2013) and transient (Soeria-Atmadja et al., 2017; Clifford et al., 2005), although there are observations of symptoms with late onset or prolonged duration lasting for months-to years as well as more severe manifestations such as seizures, suicidality and psychotic episodes (Sarfo et al., 2016; Xiao et al., 2022; Arenas-Pinto et al., 2018; van Rensburg et al., 2022). CNS symptoms associated with high plasma EFV levels and/or CYP2B6 516 TT-genotype (conferring slow metabolism) have been reported in several studies (Mukonzo et al., 2013; van Rensburg et al., 2022; Rotger et al., 2005; Haas et al., 2004), but not confirmed in others (Aouri et al., 2016; Decloedt and Maartens, 2013). *In vitro* experiments indicated that both 8-OH-EFV and 7-OH-EFV could cause neurotoxicity (Arend et al., 2016; Tovar-y-Romo et al., 2012), which a few clinical studies explored further by quantifying plasma and/or CSF EFV metabolites in adults (Aouri et al., 2016; Nightingale et al., 2016; Winston and Puls, 2014).

We recently developed and validated a liquid chromatography high-resolution tandem mass spectrometry (LC-HRMS/MS) method to quantify plasma EFV and its phase I and II metabolites in children (Pettersson et al., 2024). During analysis, a previously unknown metabolite, 6-chloro-4-[3-cyclopropyl-1-hydroxy-1-(trifluoromethyl)-2-propyn-1-yl]-2(3H)-benzoxazolone was discovered and hypothesized to be an 8-OH-efavirenz degradation product (EFAdeg). This compound was found in both patient samples and those spiked with 8-hydroxyefavirenz. We hypothesized that the formation of EFAdeg, was likely due to a hydrolysis rearrangement with a ring-opening. Ring-opening has been previously described for EFV (Maurin et al., 2002) and analogous rearrangements have been observed for the antiretroviral drug ritonavir (Rao et al., 2010). Furthermore, we proposed that the rearrangement was an equilibrium reaction, in line with our observation that 8-OH-EFV was detected in blank plasma spiked with EFAdeg (Pettersson et al., 2024).

Previously, we showed a correlation between subtherapeutic EFV levels, viremia, and the emergence of new HIV-drug resistance mutations after 6 months of treatment in a cohort of ART-naïve Ugandan children aged 3–12 years (Soeria-Atmadja et al., 2025). In the same cohort, we here describe the plasma concentrations of conjugated and unconjugated EFV, 8-OH-EFV and 7-OH as well as the newly identified compound EFAdeg and explore their association to CYP2B6 metabolizer phenotype and CNS adverse effects. Additionally, we examine potential signs of EFV autoinduction by analyzing the metabolite/EFV ratio and describe the newly identified compound EFAdeg. To our knowledge, this is the first study to quantify a selection of phase I and II EFV plasma metabolites in children.

## Materials and methods

### Study design, participants and variables monitored

This is the third publication from the prospective GENEFA cohort study (The importance of pharmacoGENetic variation on EFAvirenz levels and treatment effects in ART-naïve HIV-infected Ugandan children aged 3–12 years). The study was jointly conducted by Karolinska Institutet (KI), Sweden, Baylor College of Medicine Children’s Foundation-Uganda (Baylor Uganda) and Makerere College of Health Sciences (MakCHS), Uganda. Participants were enrolled between February 2015 and February 2016 in Baylor Uganda, an outpatient HIV facility for children and their families in Kampala. Details of the design and the cohort have been described (Soeria-Atmadja et al., 2020). Eligibility criteria included HIV-positive status, age 3–12 years, a body weight >10 kg, ART naïve status and no recent use (within 12 weeks before study start) of St. John’s wort, carbamazepine, phenytoin, phenobarbitone, or rifampicin, due to potential interactions. Out of 120 children screened, 99 were enrolled.

Adhering to Ugandan national HIV treatment guidelines, the participants were prescribed ART with EFV and two nucleotide reverse transcriptase inhibitors (NRTIs) (abacavir and lamivudine) as first-line ART, as well as co-trimoxazole prophylaxis. EFV tablets were prescribed according to WHO weight bands: 10-<14 kg/14- <25 kg/25-<35 kg/≥35 kg, with once-daily doses of 200/300/400/600 mg respectively. The NRTIs were administered as fixed drug combination tablets. Families were asked to administer the ART on an empty stomach in the evening and to report the hour of administration. Other medication that had been administered within 2 weeks before the study visit was recorded (Soeria-Atmadja et al., 2025).

Adherence to ART was assessed at each visit and estimated on a scale of 0%–100%, based on pill count (Farley et al., 2008). Adverse events were monitored by an adapted questionnaire (Supplementary Table S1) (Gounden et al., 2010), at baseline and all ensuing scheduled and unscheduled visits. It was administered by the physician to caretakers or directly to children above the age of 8 years. We asked for the presence of the following symptoms during the 2 weeks preceding the visit: headache, dizziness, nightmares/vivid dreams, hallucinations, signs of depression, confusion, difficulties concentrating, difficulty sleeping (CNS-symptoms), stomach-ache, nausea, vomiting (Gastrointestinal symptoms) and rash and how often the symptoms interfered with the child’s daily activity (“never”, “sometimes” or “most times”). The study clinician assessed and graded the severity of suspected adverse reactions according to WHO standards (World Health Organization, 2010). Mid-dose plasma concentrations of EFV and its metabolites were assessed at baseline and during follow-up at 2, 6, 12, and 24 weeks and were collected 8–20 h after the last administered dose.

### Data management, quality control, and statistical analysis

Data from standardized clinical report forms were entered and managed with REDCap (Harris et al., 2009), a secure electronic research database, following regular quality control checks. We used the software Stata version 17.0 (StataCorp LLC, Texas, United States) for analysis.

To display the relative quantity of phase I and phase II metabolites compared to EFV in plasma, we calculated metabolite/EFV ratios. Since plasma concentrations and metabolite/EFV ratios were not normally distributed the Kruskal–Wallis test was used to analyze their distribution in relation to metabolizer phenotype. This was followed by the Conover-Iman test with Holm corrections, considering a p-value <0.025 as significant.

Multivariate restricted maximum likelihood regression models (REML) were used to investigate the log_e_(metabolite/EFV), with adjustments for sex, age (years), time from treatment initiation (days), dose (mg/kg), assessed mean adherence (%) and metabolizer phenotype. Random intercepts for individuals and random slope for treatment time were employed, while the remaining explaining variables were entered as fixed effects. EFV/metabolite plasma concentrations below the lower limit of quantification were assigned a value of 0 in data analyses.

EFV autoinduction was assessed with REML which used log_e_((8-OH-EFV + EFAdeg)/EFV) as the outcome variable based on two assumptions. Firstly, a metabolite/EFV ratio based on the phase I metabolite 8-OH-EFV, was assumed to capture CYP2B6 activity more accurately than a ratio based on 8-OH-EFVtot. Secondly, 8-OH-EFV and EFAdeg were hypothesized to be in constant equilibrium and should therefore be considered together. An interaction term for metabolizer phenotype x time on treatment was added to investigate potential differences in log_e_((8OH-EFV + EFAdeg)/EFV) over time depending on metabolizer phenotype.

The Wilcoxon rank sum was used to compare the distribution of EFV and metabolite concentrations in participants with and without CNS symptoms. Plasma EFV concentration was classified as subtherapeutic if below 1,000 ng/mL, therapeutic between 1,000 and 4,000 ng/mL and supratherapeutic above 4,000 ng/mL (Marzolini et al., 2001). The χ^2^ test and Fischer’s exact test (when expected cell count was ≤5) were used for comparisons between categorical variables (differences in proportions).

### Analysis of efavirenz and metabolite plasma concentrations

Mid-dose plasma samples for analysis of EFV and metabolites were collected in lithium-heparin tubes before ART-start and 2, 6, 12 and 24 weeks thereafter The median time from self-reported dosing to sampling was 12.9 h (Soeria-Atmadja et al., 2025). Samples were stored at −80 °C. A LC-HRMS/MS method, developed and validated by the Department of Clinical Pharmacology at Karolinska University Hospital, was used to detect and quantify efavirenz and its phase I and II metabolites as described (Pettersson et al., 2024).

In short, samples were prepared by protein precipitation of 100 µL plasma and then divided into two different aliquots. The first supernatant aliquot was prepared without enzymatic hydrolysis and was used to quantify (unconjugated) EFV and phase I metabolites (7-OH-EFV and 8-OH-EFV). A second aliquot underwent hydrolyzation mediated by β-glucuronidase/arylsulfatase to quantify the total (sum of conjugated and unconjugated) concentration of each metabolite: EFV-tot (the concentration of EFV + EFV-N-glucuronide), 8-OH-EFV-tot (the concentration of 8-OH-EFV+ 8-OH-EFV-glucuronide/sulfate) and 7-OH-EFV-tot (the concentration of 7-OH-EFV + 7-OH-glucuronide/sulfate) (Figure 1).

We also quantified the newly identified compound EFAdeg (6-Chloro-4-[3-cyclopropyl-1-hydroxy-1- (trifluoromethyl)-2-propyn-1-yl]-2(3H -benzoxazolone) in plasma. The plasma concentrations were measured for unconjugated EFAdeg, and for the total concentration of EFAdeg + EFAdeg-conjugates (EFAdeg-tot), as described for EFV and 7-OH-EFV and 8-OH-EFV. The phase II metabolites of 8-OH-EFV, 7-OH-EFV and EFAdeg were not individually quantified, as the method could not differentiate between the contribution from glucuronidated and sulfated substances. The analytes were chromatographically separated on a Dionex Ultimate 3000RS UHPLC system (Thermo Fisher Scientific, Waltham, United States) using an Hypersil Gold RP C18 column (50 × 2.1 mm, 1.9 µm, Thermo Fisher Scientific) operating at 50 °C. Mobile phases consisted of 10 mmol/L aqueous ammonium formate plus 0.015% formic acid (A) and methanol (B) and the flow rate was 0.4 mL/min. Analyte detection was performed using a Q-Exactive system (Thermo Fisher Scientific) equipped with a heated electrospray ionization (HESI) source. MS detection was performed in both full MS and in PRM mode using negative electrospray mode. The quantification transitions for EFV and its phase I metabolites were 314.0201 > 244.0167 (EFV), 330.0150 > 257.9939 (7-OH-EFV), 330.0150 > 266.0193 (8-OH-EFV) and 330.0150 > 216.0215 (EFAdeg) (Pettersson et al., 2024). Concentrations were converted from molar units (mol/L) to mass units (ng/mL) for reporting purposes. The range of measurement was 100–50 000 ng/mL for EFV, EFV-tot, 8-OH-EFV, and 8-OH-EFV-tot, 125–25,000 ng/mL for 7-OH-EFV, and 7-OH-EFV-tot, and 200–10,000 ng/mL for EFAdeg and EFAdeg-tot.

### Genotyping and classification of metabolizer phenotype

As described (Soeria-Atmadja et al., 2025), the following polymorphisms in the CYP2B6 and CYP2A6, genes were analyzed: CYP2A6 g.-48T>G (rs28399433, C_30634332_10), CYP2B6 c.516G>T (rs3745274, C_7817765_60) and CYP2B6 c.983T>C (rs28399499, C_60732328_20). Participants were categorized according to their composite genotype of the two SNPs CYP2B6 c.516G>T and CYP2B6 c.983T>C and thereafter assigned a predicted metabolizer phenotype as extensive metabolizer (EM), 516GG|983TT, intermediate metabolizer (IM), 516GG|983TC or 516GT|983TT, slow metabolizer (SM) 516GT|983TC or 516TT|983TT as described (Dooley et al., 2015; Bienczak et al., 2016). No ultraslow metabolizer (USM), 516GG|983CC was identified in our population (Soeria-Atmadja et al., 2025). Rapid and ultrarapid metabolizer phenotypes (Desta et al., 2019) were not assessed in this study.

### Ethics

The study was approved by the Institutional Review Board of School of Biomedical Sciences and Higher Degrees, Makerere University College of Health Sciences (SBS-HDREC 174), Uganda National Council for Science and Technology UNCST (HS1659), Baylor College of Medicine Children’s Foundation IRB Texas (H35946) and the Regional Ethical Review Board in Stockholm, Sweden (2016/1026-31). Written informed consent/assent was obtained from caretakers/children. Treatment and services offered followed the standard clinical procedures at Baylor Uganda.

## Results

### Baseline characteristics

Baseline characteristics of our cohort (99 children) were previously described, with a median age of 6 years, and a female/male ratio of 59.6/40.4% (Soeria-Atmadja et al., 2020). The distribution of the metabolizer phenotypes predicted from the composite genotypes of CYP2B6 c.516G>T and CYP2B6 c.983T>C for the whole cohort was 28.9% EM, 55.7% IM and 15.5% SM. For the SNP CYP2A6 g.-48T>G, 90% of participants were classified as wild type carriers, 9% and 1% as heterozygous and homozygous carriers, respectively (Soeria-Atmadja et al., 2025).

### Efavirenz and metabolites plasma concentrations

Adherence was previously described (Soeria-Atmadja et al., 2025) and mean adherence per visit ranged from 96% to 97% (range 52%–100%) and did not differ by sex, age, or metabolizer phenotype, but was significantly lower in individuals with subtherapeutic EFV plasma concentrations (Soeria-Atmadja et al., 2025).

**FIGURE 2:**
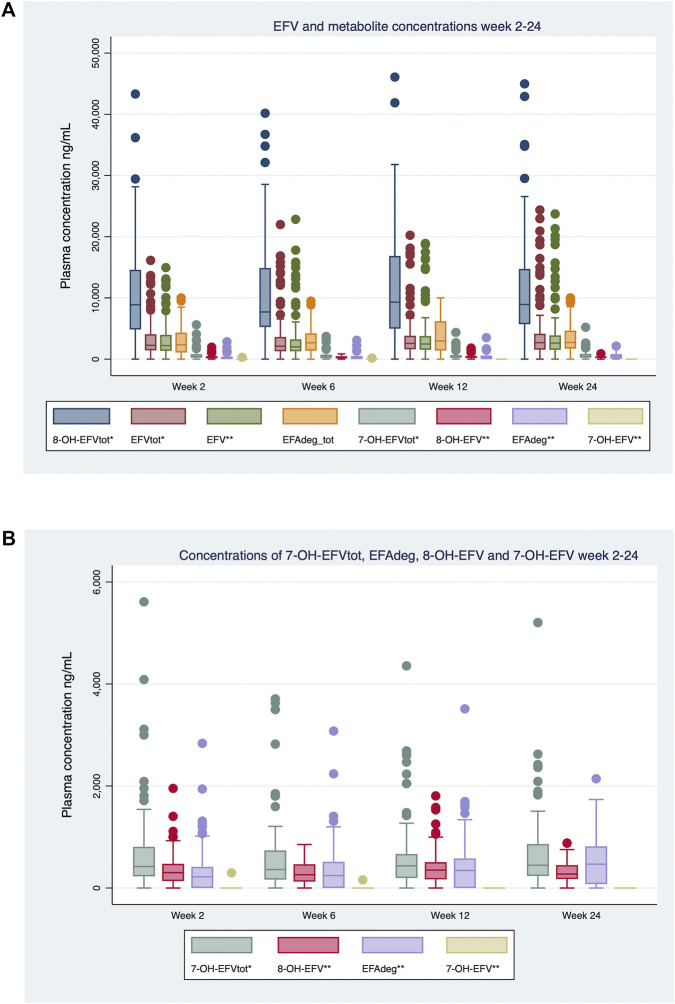
Ninety-nine ART-naive Ugandan children aged 3-12 years were enrolled and initiated EFV-based antiretroviral therapy. Mid-dose efavirenz and metabolites plasma concentrations (ng/ml) were sampled at 2, 6, 12 and 24 weeks. *Total concentration of unconjugated and conjugated substance. **Concentration for unconjugated substance only. Number of successful measurements per analyte and visit for week 2, 6, 12, 24: 8-OH-EFV-tot 88, 92, 87, 92; EFV-tot 95, 91, 92, 94; EFV 95, 94, 92, 94; EFAdeg-tot 87, 91, 88, 92; 7-OH-EFV-tot 95, 93, 92,94; EFAdeg 87, 91, 88, 92; 8-OH-EFV 89, 92, 87, 92; 7-OH-EFV 95, 93, 92, 94. Line inside the box denotes the median, while lower and upper box boundaries represent the 25th and 75th percentiles, respectively. Data points more than 1,5 box-lengths away from 25th or 75th percentiles are represented by dots. **(A)** Plasma concentrations of efavirenz and all measured metabolites. **(B)** Metabolites in **(A)** with low plasma concentrations (7-OH-EFV, 7-EFV-OHtot, 8-OH-EFV and EFAdeg) displayed with an adjusted y-axis scale.

Baseline plasma samples before treatment start were available for all 99 participants. All had undetectable plasma concentrations of EFV and its metabolites, except for two individuals with 8-OH-EFV-total concentrations of 204 and 186 ng/mL, respectively. The distribution of EFV and metabolite plasma concentrations at weeks 2, 6, 12 and 24 is shown in Figure 2 and Supplementary Table S2.

Metabolite/EFV ratios, are displayed in Supplementary Table S3. For the entire cohort, 8-OH-EFV-tot (including both unconjugated and conjugated forms) was predominant, followed by EFAdeg-tot (conjugated and unconjugated), as the second most common metabolite. Throughout the study, the unconjugated 8-OH-EFV and EFAdeg accounted for a limited amount of the total metabolite concentrations (8-OH-EFV-tot and EFAdeg-tot). (Figure 2) (Supplementary Tables S2 and S3). 7-OH-EFV was only quantifiable in two samples (two SM individuals). The EFV-N-gln constituted only a minor part of circulating EFV, the median EFV-tot/EFV unconjugated ratio being 1:1.

### The influence of CYP2B6 metabolizer phenotypes and other covariates on efavirenz and metabolites

**FIGURE 3:**
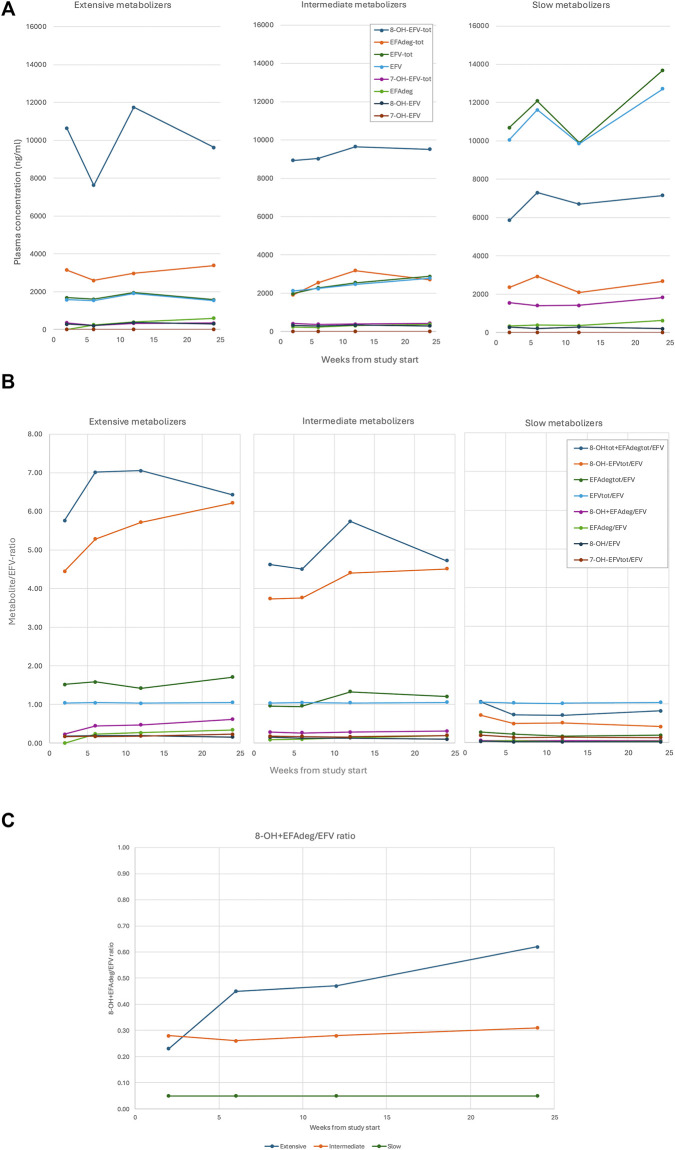
Efavirenz and metabolites plasma concentrations and metabolite: EFV ratios by metabolizer type. Ninety - seven Ugandan children aged 3–12 years, were classified as extensive, intermediate or slow metabolizer phenotype based on composite genotype of CYP2B6 516G>T/983T>C, with 28, 54 and 15 children in each group. **(A)** Median plasma concentration of EFV and metabolites (ng/mL) per visit and metabolizer type, by weeks from therapy start. 8-OH-EFVtot (8-OH-EFV + 8-OH-EFV-sulfate+ 8-OH-glucuronide), 7-OH-EFVtot (7-OH-EFV + 7-OH-EFV-sulfate + 7-OH-glucuronide), EFV-tot (EFV + EFV-N-glucuronide), EFAdeg-tot (EFAdeg + EFAdeg-conjugate). **(B)** Metabolite: EFV ratio, median per visit and metabolizer type, by weeks from therapy start. EFAdeg was hypothesized to be an 8-OH-EFV degradation product in equilibrium with 8-OH-EFV, why metabolite: EFV ratio was calculated also for the combined concentrations of 8-OH-EFV and EFAdeg and the combined concentrations of 8-OH-EFV-tot + EFAdeg-tot. **(C)** 8-OH-EFV + EFAdeg: EFV ratio, median per visit and metabolizer type, by weeks from therapy start.

Plasma concentrations of EFV and metabolites by metabolizer phenotypes at weeks 2, 6, 12 and 24 are displayed in Figure 3A and Supplementary Table S4 and the metabolite/EFV ratios throughout the study period in Figure 3B, Supplementary Table S5. The distribution of metabolite/EFV ratio for 8-OH-EFV + EFAdeg is displayed in Figure 3C.

The median metabolite/EFV ratios differed significantly across CYP2B6 metabolizer phenotypes for 8-OH-EFV-tot, EFAdeg-tot, and 8-OH-EFV (p-<0.0001–0.0097) at all study visits and SM consistently showed lower ratios than IM and EM in pairwise testing (Supplementary Table S5). A similar pattern was seen for EFAdeg, except at week 2, when no significant difference was observed among metabolizer phenotypes (Supplementary Table S5). The median metabolite/ratios for EFAdeg-tot and 8-OH-EFV-tot were significantly higher in extensive than intermediate metabolizers at all visits except week 12 (Supplementary Table S5).

The multivariate REML model assessing longitudinal changes in CYP2B6 metabolic capacity identified metabolizer phenotype as a strong predictor of log_(e)_ ((8-OH-EFV + EFAdeg)/EFV). The log_(e)_metabolite/EFV-ratio was significantly higher in IM (coefficient = 1.7) and EM (coefficient = 2.3) compared to SM (coefficient = −2) (p < 0.0001) and differed significantly between EM and IM (p = 0.003) (Table 1).

**TABLE 1:** Predictors of log_(e)_ ((8-OH-EFV + EFAdeg)/EFV) in a multivariate restricted maximum likelihood regression (REML) model.

Log_(e)_ ((8-OH-EFV + EFAdeg)/EFV)	Model without interaction variable			​	Model with interaction variable			​
	Coefficient	P > z	[95% conf.	Interval]	Coefficient	P > z	[95% conf.	Interval]
Metabolizer phenotype#time on treatment	—	—	—	—	​	​	​	​
Intermediate	—	—	—	—	0.0019	0.2550	−00014	0.0052
Extensive	—	—	—	—	0.0039	**0.0370**	0.0002	0.0077
Time on treatment (days)	0.0008	0.1970	−0.0004	0.0020	−0.0013	0.3700	−0.0042	0.0016
Age at treatment start (years)	0.0713	**0**.**0360**	0.0045	0.1380	0.0733	**0.0310**	0.0069	0.1398
Sex (female)	−0.1794	0.2760	−0.5021	0.1432	−0.1684	0.3040	−0.4895	0.1527
EFV dose (mg/kg)	−0.0519	0.0760	−0.1093	0.0055	−0.0505	0.0840	−0.1078	0.0068
Mean_Adherence (%)	−0.0070	0.7140	−0.0445	0.0305	−0.0080	0.6760	−0.0453	0.0294
Metabolizer phenotype	​	​	​	​	​	​	​	​
Intermediate	1.7441	**<0**.**0001**	1.2980	2.1902	155995	**<0**.**0001**	1.0876	2.1115
Extensive	2.2811	**<0**.**0001**	1.7832	2.7790	1.9687	**<0**.**0001**	1.3940	2.5434
_Constant	−1.9980	0.3030	−5.8017	1.8056	−1.7855	0.3560	−5.5759	2.0048

Ninety-nine ART-naive Ugandan children aged 3–12 years initiated efavirenz (EFV)-based antiretroviral therapy and were classified into extensive (n = 28), intermediate (n = 54), or slow metabolizer phenotypes (n = 15) based on composite CYP2B6 516G>T/983T>C genotype. Mid-dose EFV and its metabolite plasma concentrations (ng/mL) were measured at 2, 6, 12, and 24 weeks. As EFAdeg and 8-OH-EFV were hypothesized to be in equilibrium, the ratio 8-OH-EFV + EFAdeg/EFV was used to estimate of CYP2B6 activity. Predictors of CYP2B6 activity was investigated in a multivariate REML with log_(e)_ (8-OH-EFV + EFAdeg/EFV as an outcome variable. The analysis included 292 observations of 8-OH-EFV + EFAdeg/EFV in 92 participants (in average 3,2 observations per individual). Reference groups for categorical variables were “male” and “slow metabolizer phenotype”. Statistically significant p-values appear in bold.

Similarly, metabolizer phenotype predicted log_(e)_metabolite/EFV ratios for 8-OH-EFV, EFAdeg, 8-OH-EFV-tot, EFAdeg-tot in multivariate REML models (Supplementary Table S6).

In contrast, median plasma concentrations of 7-OH-EFV-tot were significantly higher in slow metabolizers compared with intermediate and extensive metabolizers at all visits (p < 0.0001) (Supplementary Table S4), a finding confirmed in the corresponding multivariate REML model investigating predictors of log_(e)_7-OH-EFV-tot (p < 0.0001) (Supplementary Table S6). The median EFV-tot/EFV ratio did not differ significantly between metabolizer groups (Supplementary Table S5).

Female participants exhibited lower log_(e)_metabolite/EFV ratios for both 8-OH-EFV-tot (coefficient = −0.48, p = 0.005) and EFAdeg-tot, (coefficient = −0.32, p = 0.045) (Supplementary Table S6). Gender had no significant effect on any other log_(e)_metabolite/EFV ratio. Except for the combined 8-OH-EFV + EFAdeg ratio (see below), age was not associated with changes in log_(e)_metabolite/EFV ratios (Supplementary Table S6). Duration of therapy was significantly associated with a lower log_(e)_8-OH-EFV/EFV ratio but did not influence other metabolite/EFV ratios (Supplementary Table S6).

### Estimation of EFV autoinduction using log_(e)_ ((8-OH-EFV + EFAdeg)/EFV)

The results of a multivariate REML showed a significant positive effect of age on log_(e)_ ((8-OH-EFV + EFAdeg)/EFV) while treatment duration did not significantly affect the outcome variable (Table 1). An interaction term (days on treatment x metabolizer phenotype) was added to the REML model, to evaluate if time-dependent change for log_(e) _((8-OH-EFV + EFAdeg)/EFV) -i.e. the slope for “time on treatment (days)”-would vary according to metabolizer phenotype. The slopes were estimated to 0.0026, 0.0006 and −0.0013 respectively for EM, IM and SM, but only the slope for EM was significantly different from 0 (p = 0.027). Pairwise comparisons also indicated different slopes for EM and SM (p = 0.037). Thus, according to the REML model, the log_(e) _((8-OH-EFV + EFAdeg)/EFV) significantly increased over time exclusively among EM, pointing towards EFV autoinduction specific to this group. Figure 3C illustrates the median (8-OH-EFV + EFAdeg)/EFV ratios per visit and metabolizer phenotype.

### 7-OH-EFV plasma concentration and metabolic ratio by CYP2A6 g.-48T>G genotype

We investigated the distribution of plasma concentrations and metabolite/EFV ratios for 7-OH-EFV and 7-OH-EFV-tot in relation to the CYP2A6 g.-48T>G genotypes. One CYP2B6 extensive metabolizer was homozygous for CYP2A6 g.-48T>G (and had undetectable levels of both 7-OH-EFV and 7-OH-EFV-tot), while 2 extensive, 6 intermediate and 1 slow CYP2B6 metabolizers were heterozygous carriers of the G-allele. To explore the potential impact of CYP2A6 g.-48T>G we compared the GT/GG genotypes (n = 10) with those who carried the TT genotype (n = 87) and found no difference in median plasma concentration or metabolite/EFV ratio for 7-OH-EFV. For 7-OH-EFV-tot, the median plasma concentration only differed significantly between the TT genotype (508 ng/mL) and the GT/GG genotypes (201 ng/mL) at week 24 (p = 0.029). The 7-OH-EFV-tot/EFV ratio was significantly lower in the GT/GG group vs. the TT group at visit 6, 12 and 24 with ratios of 0.11 vs. 0.17 (visit 6, p = 0.01), 0.11 vs. 0.17 (visit 12, p = 0.03) and 0.08 vs. 0.21 (visit 24, p = 0.006).

### Adverse events

**FIGURE 4:**
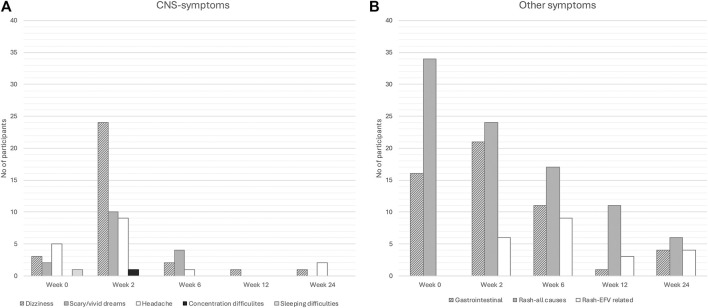
Adverse events. Before starting antiretroviral therapy (ART) at week 0 and during the subsequent four visits, participants reported adverse drug reactions (ADRs) using a questionnaire, that screened for CNS-symptoms **(A)** and other symptoms **(B)**. The number of participants at each time point was 99, 97, 95, and 94 for weeks 0, 2, 6, 12, and 24, respectively. From week 2 to week 24, a study clinician evaluated and graded potential ADRs. For central nervous system (CNS) and gastrointestinal symptoms, the clinician’s assessment of ADRs aligned with the participants’ reports. However, many of the rashes reported by participants were not considered adverse reactions by the clinician. The graph **(B)** illustrates both the total number of rashes reported by participants (“Rash - all causes”) and those assessed as potentially efavirenz (EFV)-related by the clinician (“Rash - EFV”). Gastrointestinal symptoms included nausea, abdominal pain, and vomiting.

The adverse events reported by participants throughout the study are displayed in Figure 4. Before treatment start, a total of 34% (34/99) participants experienced rash, while symptoms from the CNS and gastrointestinal tract were reported by 11% (11/99) and 16% (16/99) individuals respectively. The symptoms reported after therapy start peaked at week 2, subsequently declined and were throughout the study, classified as either mild (96%) or moderate (4%) by the clinician. Participants/caregivers graded the symptoms as “never” (62%), “sometimes” (32%) or “most times” (6%) interfering with the child’s daily activity.

Thirty-six individuals reported at least one or more CNS-symptoms (dizziness, headache, vivid/scary dreams, sleeping difficulties and concentration difficulties) from week 2 and onwards. Among them, 33% (12/36) exhibited individual median EFV plasma concentration above the therapeutic interval, compared to only 15% (9/61) of participants without CNS symptoms (p = 0.032). Similarly, the occurrence of any CNS symptoms up to week 24 were more prevalent among SM (60%) compared to EM and IM (33%),(p = 0.046).

However, CNS symptoms reported at individual visits were not significantly associated with either supratherapeutic EFV plasma concentrations measured at the same visit or the CYP2B6 metabolizer phenotype (Table 2). Similarly, participants who reported CNS symptoms between weeks 2 and 24 showed no statistically significant differences in median plasma concentrations of EFV or its phase I or phase II metabolites at any visit, compared with participants without CNS symptoms. (Table 3).

**TABLE 2:** CNS symptoms in relation to supratherapeutic EFV plasma concentrations and CYP2B6 metabolizer type, shown by week of testing and for the whole study period combined.

EFV plasma concentration					​	CYP2B6 metabolizer phenotype			
Week 2	​	Supratherapeutic (>4,000 ng mL)	Therapeutic-subtherapeutic (≤4,000 ng mL)	​	p-value	Slow	Intermediate/Extensive	​	p-value
CNS-symptoms	​	9 (39.1%)	20 (27.8%)	29	​	8 (46.7%)	21 (25.6%)	29	​
No CNS-symptoms	​	14 (60.9%)	52 (70.2%)	66	​	7 (53.3%)	61 (74.4%)	68	​
​	**n**	23	72	95	0.30	15	82	97	**0.031**
Week 6	​	​	​	​	​	​	​	​	​
CNS-symptoms	​	4 (20%)	5 (6.8%)	9	​	7 (35%)	2 (2.7%)	9	​
No CNS-symptoms	​	16 (80%)	69 (93.2%)	85	​	13 (65%)	73 (97.3%)	86	​
​	**n**	20	74	94	0.093*	20	75	95	0.63*
Week 12	​	​	​	​	​	​	​	​	​
CNS-symptoms	​	1 (4.8%)	0	1	​	0	1 (1.3%)	1	​
No CNS-symptoms	​	20 (95.2%)	71 (100%)	91	​	15 (100%)	78 (98.7%)	93	​
​	**n**	21	71	92	1.00*	15	79	94	​
Week 24	​	​	​	​	​	​	​	​	1.00*
CNS-symptoms	​	1 (4.5%)	2 (2.8%)	3	​	0	3 (3.8%)	3	​
No CNS-symptoms	​	21 (95.5%)	70 (97.2%)	91	​	15 (100%)	76 (96.2%)	91	​
​	**n**	22	72	94	0.56*	15	79	94	1.00*

Individual EFV median concentration week 2–24					​	CYP2B6 metabolizer phenotype			
​	​	Supratherapeutic (>4,000 ng mL)	Therapeutic-subtherapeutic (≤4,000 ng mL)	​	p-value	Slow	Intermediate/Extensive	​	p-value
CNS-symptoms	​	12 (57%)	24 (31.6%)	36	​	9 (60%)	27 (32.9%)	36	​
No CNS-symptoms	​	9 (43%)	52 (68.4%)	61	​	6 (40%)	55 (67.1%)	61	​
​	**n**	19	76	97	**0.032**	15	82	97	**0.046**

Association between central nervous system (CNS) symptoms, efavirenz (EFV) plasma concentration categories, and CYP2B6 metabolizer phenotype - defined by the composite genotype of CYP2B6 c.516G>T and c.983T>C - in Ugandan children at weeks 2, 6, 12, and 24, and across the combined study period. Data are presented as number (%). Group comparisons were performed using the χ^2^ (chi-square) test or Fisher’s exact test*. Statistically significant p-values are displayed in bold.

**TABLE 3:** CNS symptoms and EFV plasma concentrations (ng/mL).

Week and analyte	No CNS-symptoms		CNS-symptoms	​
​	Median plasma concentration	IQR	Median plasma concentration	IQR	p-value
**Week 2**	(n = 68)	​	(n = 29)	​	​
EFV	2,153	(1,358–3,549)	3,053	(1,441–5,594)	0.30
EFV-tot	2,181	(1,406–3,712)	3,196	(1,472–5,774)	0.29
8-OH-EFV	296	(121–405)	355	(192–639)	0.089
8-OH-EFV-tot	6,980	(4,940–13,405)	10,965	(4,756–20,086)	0.26
EFAdeg	0	(0–395)	280	(0–553)	0.16
EFAdeg-tot	2,334	(1,079–3,878)	2,130	(1,218–4,915)	0.64
EFAdeg + 8-OH-EFV	409	(121–789)	604	(195–1,255)	0.089
EFAdeg + 8-OH-EFV-tot	10,319	(6,054–16,490)	13,287	(5,975–21,645)	0.46
7-OH-EFV	0	(0–0)	0	(0–0)	0.13
7-OH-EFV-tot	370	(208–701)	644	(381–1,087)	0.054
**Week 6**	(n = 86)	​	(n = 9)	​	​
EFV	1,945	(1,290–3,009)	3,793	(1,283–5,294)	0.63
EFV-tot	2,095	(1,329–3,291)	4,045	(1,244–5,545)	0.69
8-OH-EFV	248	(122–476)	382	(211–457)	0.47
8-OH-EFV-tot	7,784	(5,249–16,754)	7,217	(5,053–9,006)	0.21
EFAdeg	244	(0–524)	223	(0–295)	0.49
EFAdeg-tot	2,679	(1,391–4,403)	2,543	(1,274–3,285)	0.46
EFAdeg + 8-OH-EFV	543	(161–985)	434	(282–823)	0.83
EFAdeg + 8-OH-EFV-tot	10,432	(7,096–20,324)	10,502	(6,327–12,807)	0.32
7-OH-EFV	0	(0–0)	0	(0–0)	0.76
7-OH-EFV-tot	363	(165–754)	368	(146–524)	0.80
**Week 12**	(n = 93)	​	(n = 1)	​	​
EFV	2,460	(1,518–3,766)	2,486	-	0.98
EFV-tot	2,543	(1,591–3,832)	2,799	-	0.87
8-OH-EFV	342	(169–482)	905	-	0.16
8-OH-EFV-tot	9,298	(4,976–16,855)	11,346	-	0.84
EFAdeg	343	(0–579)	1,625	-	0.096
EFAdeg-tot	3,027	(1,411–6,448)	3,819	-	0.68
EFAdeg+8-OH-EFV	709	(297–1,099)	2,530	-	0.13
EFAdeg+8-OH-EFV-tot	12,114	(6,615–24,536)	15,165	-	0.75
7-OH-EFV	0	​	0	-	-
7-OH-EFV-tot	445	(198–695)	0	-	0.13
**Week 24**	(n = 91)	​	(n = 3)	​	​
EFV	2,612	(1,436–3,875)	2,796	(1,648–4,570)	0.80
EFV-tot	2,687	(1,519–4,141)	2,947	(1,689–4,664)	0.79
8-OH-EFV	275	(173–449)	336	(272–380)	0.53
8-OH-EFV-tot	8,943	(5,841–14,721)	6,000	(4,967–14,737)	0.55
EFAdeg	453	(0–766)	1,300	(271–1,397)	0.21
EFAdeg-tot	2,739	(1,809–4,656)	3,065	(1,925–6,405)	0.72
EFAdeg + 8-OH-EFV	828	(301–1,202)	1,572	(607–1,776)	0.21
EFAdeg + 8-OH-EFV-tot	12,282	(8,127–19,858)	9,065	(6,893–21142)	0.68
7-OH-EFV	0	(0–0)	0	(0–0)	-
7-OH-EFV-tot	442	(237–863)	583	(281–1,065)	0.69

Median (interquartile range, IQR) plasma concentrations (ng/mL) of efavirenz (EFV) and its metabolites in 99 antiretroviral therapy–naïve Ugandan children (aged 3–12 years) at weeks 2, 6, 12, and 24 after initiation of EFV-based therapy. Unconjugated analytes (EFV, 7-OH-EFV, and 8-OH-EFV) and total concentrations (unconjugated plus conjugated forms: EFV-tot, 7-OH-EFV-tot, and 8-OH-EFV-tot) are shown. EFV-tot includes EFV and EFV-N-glucuronide; 7-OH-EFV-tot includes sulfate and glucuronide conjugates; and 8-OH-EFV-tot includes sulfate and glucuronide conjugates. EFAdeg was hypothesized to be a degradation product of 8-OH-EFV. CNS symptoms were assessed at each visit, and group differences were evaluated using the Wilcoxon rank-sum test (p < 0.05). Concentrations below the lower limit of quantification were set to zero for analysis.

There were no reports of severe psychiatric symptoms such as hallucinations, suicidality, or confusion nor reports of hospitalization or treatment discontinuation due to EFV-related adverse events. The CNS-symptoms were reversible for most participants - only two (both IM) reported continued CNS-symptoms (headache and dizziness irrespectively) beyond week 12.

## Discussion

We conducted a study in ART naïve Ugandan children with plasma concentration measurements of EFV and its phase I and II metabolites, over 24 weeks. To the best of our knowledge, this is the first study to quantify EFV metabolites in children. Furthermore, we describe the plasma concentration of a recently identified substance, EFAdeg, that may be a degradation product of 8-OH-EFV (Pettersson et al., 2024) in relation to other EFV metabolites.

The EFV phase I metabolites 8-OH-EFV and 7-OH-EFV were detected in low concentrations in plasma, compared to phase II metabolites, which is consistent with findings in adults (Cho et al., 2011; Aouri et al., 2016; Avery et al., 2013b). In individuals categorized as either CYP2B6 extensive or intermediate metabolizers, the main circulating substance was glucuronidated/sulfated 8-OH-EFV. Among CYP2B6 slow metabolizers, the parent drug EFV was the predominant substance, and the *total* 7-OH-EFV plasma concentration (conjugated and unconjugated) was significantly elevated compared to EM and IM (p < 0.0001). In addition, (unconjugated) 7-OH-EFV was only detected in two SM. This likely reflects that SM is more dependent on the alternative metabolic pathway of CYP2A6 to compensate for poor CYP2B6 activity, as previously shown in adults. The low levels of unconjugated 7-OH-EFV among participants in general, is in line with previous reports (Ogburn et al., 2010; Aouri et al., 2016; Avery et al., 2013b) suggesting that CYP2A6 accounts for a minor part of EFV hydroxylation and that the low amount of 7-OH-EFV formed, is rapidly further metabolized *via* glucuronidation/sulfation (Aouri et al., 2016). Throughout the study, EFV-N-gln/EFV ratio remained close to 1 across all three metabolizer groups indicating that very little EFV undergoes N-glucuronidation, as described in adults (Cho et al., 2011).

In adults, the plasma levels of 7-OH-EFV and 8-OH-EFV glucuronides are higher than the plasma concentrations of 8-OH-EFV-sulfate and 7-OH-EFV-sulfate (Aouri et al., 2016). Several isoforms of UDP-glucuronosyltransferases (UGTs) such as UGT1A1, UGT1A3, UGT1A6, and UGT1A9 are believed to be involved in glucuronidation of 8-OH-EFV and 7-OH-EFV (Bae et al., 2011) and are also proposed to have very low activity levels in neonates and infants, gradually increasing throughout childhood (Bhatt et al., 2019). Consequently, children could be expected to display lower plasma levels of glucuronidated EFV hydroxy metabolites than adults. However, we were unable to investigate this as the relative contributions of the glucuronidated and sulfated metabolites, could not be determined. Interestingly, girls seemed to have lower metabolite/EFV ratios for both 8-OH-EFV-tot and EFAdeg-tot from week 6 and onwards, which could imply a gender difference in glucuronidation and/or sulfation capacity, as has been suggested for other drugs (Schwartz, 2003).

We recently reported a novel substance (Pettersson et al., 2024), which we hypothesize is a degradation product of 8-OH-EFV (EFAdeg), mediated by hydrolysis. Whether this hydrolysis occurs within the body or after sampling is unknown. However, we doubt that EFAdeg formation is due to degradation from prolonged sample storage of 8-OH-EFV, as EFAdeg was also detectable in freshly collected plasma from a patient (Pettersson et al., 2024). The distribution of EFAdeg and EFAdeg-tot followed the pattern of 8-OH-EFV, with elevated levels in CYP2B6 intermediate and extensive metabolizer compared to slow metabolizers. The clinical importance is unclear and in this study, we found no association between EFAdeg plasma concentration and the occurrence of CNS-symptoms. However, EFAdeg could inadvertently influence the measurements of 8-OH, if using a method that is not specific enough.

The 8-OH-EFV/EFV ratio has been used as a phenotypic marker of CYP2B6 (Cho et al., 2011; Aouri et al., 2016; di Iulio et al., 2009; Decloedt et al., 2015). We hypothesized that the ratio between the sum of 8-OH-EFV and EFAdeg and EFV ((8-OH-EFV + EFAdeg)/EFV) might be the most suitable phenotypic index in our cohort, based on the theory that EFAdeg and 8-OH-EFV are in equilibrium (Pettersson et al., 2024) and that both represent phase I metabolism. This ratio was significantly different between the three metabolizer groups. In the multivariate REML model, no evidence was found for overall changes in this ratio over time. However, when examining the interaction between treatment duration and metabolizer phenotype, a small but significant increase in the (8-OH-EFV + EFAdeg)/EFV ratio over time was observed specifically in extensive metabolizers (EM). This finding may indicate EFV autoinduction, consistent with previous reports in extensive metabolizers (Habtewold et al., 2011; Ngaimisi et al., 2010; Ngaimisi et al., 2011).

The CYP2A6 g.-48T>G polymorphism may reduce CYP2A6 activity, potentially elevating EFV plasma levels in CYP2B6 slow metabolizers, who are more dependent on this accessory pathway compared to intermediate/extensive metabolizers (Soeria-Atmadja et al., 2017; Haas et al., 2014). We previously (Soeria-Atmadja et al., 2025) found no association between this SNP and EFV plasma levels, likely because only one CYP2B6 slow metabolizer carried the variant, while the remaining carriers were intermediate or extensive metabolizers. In the current analysis, CYP2A6 g.-48T>G wildtype individuals exhibited a higher 7-OH-EFVtot/EFV ratio than heterozygous/homozygous carriers from week 6 onwards, even among CYP2B6 intermediate and extensive metabolizers. Although this implies that CYP2A6 g.-48T>G impairs CYP2A6 function, the clinical significance may be negligible in intermediate/extensive CYP2B6 metabolizers, as previously suggested (di Iulio et al., 2009). Further, these findings should be interpreted with caution, as 7-OH-EFVtot reflects both phase I and II metabolism and may be influenced by factors not accounted for.

The frequency of adverse events was high and peaked at week 2 when nearly one-fourth of the participants reported dizziness. Overall, more than one-third experienced CNS-symptoms at some point after therapy start, consistent with previous pediatric EFV-studies reporting CNS and neuropsychiatric symptoms in between 18% and 60% of cases (Soeria-Atmadja et al., 2017; Van de Wijer et al., 2019; Wynberg et al., 2018; Tukei et al., 2012; Shubber et al., 2013; Teglas et al., 2001). Notably, two-thirds of CYP2B6 slow metabolizers reported CNS-symptoms compared to one-third of intermediate and extensive metabolizer phenotypes.

Rare but severe neuropsychiatric and CNS manifestations such as hallucinations, confusion, depression, ataxia and seizures as described in other pediatric studies (Pinillos et al., 2016; Puthanakit et al., 2009; Hammond et al., 2019; Hauptfleisch et al., 2015), were absent in our cohort, where clinical symptoms in general were mild and declined within 12 weeks of treatment. Possibly, this is due to a relatively short period of follow-up. In adults, late onset cases of EFV-related ataxia and encephalopathy have been reported (van Rensburg et al., 2022; Variava et al., 2017), typically occurring in CYP2B6 slow metabolizers with very high plasma levels of EFV and with symptoms presenting after a median of 2 years of treatment (van Rensburg et al., 2022).

In our cohort, the presence of CNS side effects was not significantly associated with efavirenz plasma concentrations at individual time points. However, children with supratherapeutic efavirenz concentrations sustained across multiple visits were significantly more likely to report CNS symptoms. This discrepancy may be explained by intraindividual variability in EFV plasma levels, delayed or persistent CNS effects, and underlying pharmacogenetic differences that influence both plasma levels and susceptibility to side effects. Disease related manifestations or other factors such as the concomitant use of other medications (Soeria-Atmadja et al., 2025), could have contributed to the early reports within the first 6 weeks. The decline in the number of ADR reports over time may also be due to participants developing tolerance to the drug.

In contrast to EFV, we could not link the occurrence of CNS-symptoms to the plasma levels of 7-OH-EFV, 8-OH-EFV or their phase II metabolites. This aligns with findings in adults where no significant difference in plasma and CSF levels of 7-OH-EFV, 8-OH-EFV or phase II metabolites were observed between those discontinuing EFV therapy due to CNS-symptoms and those stopping for other reasons (Aouri et al., 2016). Similarly, there was no correlation found between plasma/CSF levels of 7-OH-EFV, 8-OH-EFV and neurocognitive impairment in South African adults (Decloedt et al., 2019). Others found that levels of 8-OH-EFV in CSF correlated with sleep disturbances and drowsiness and that plasma 8-OH-EFV levels were associated with mood changes (Grilo et al., 2016; Ranzani et al., 2024), in support of *in vitro* findings implicating EFV, 7OH-EFV and 8OH-EFV as potential neurotoxic agents with 8-OH-EFV being the most potent inducer of dendritic injury (Arend et al., 2016; Tovar-y-Romo et al., 2012).

The strengths of this study include the prospective design, the repeated EFV metabolite measurements, a relatively large pediatric cohort and a low rate of loss-to-follow-up. The study scope was comprehensive and addressed both pharmacogenetics and clinical effects in relation to EFV metabolites. The relatively short follow-up time is a limitation. Another limitation is that the ADR questionnaire had not been tested and validated for children. Applying specific questions regarding adverse effects could lead to overreporting, and disease-related symptoms could unintentionally be reported as adverse effects. Yet, it allows systematic collection of data over time and a rough understanding of the type, severity and duration of the adverse effects in children on treatment. The medication was administered at home, and the drugs may not have been taken as advised. The levels of conjugated substances in plasma are potentially affected by factors not controlled for in our study, such as genetic variability in phase II enzymes or renal clearance. We did not measure metabolites in urine or in CSF which could have contributed to a deeper understanding of EFV metabolism and possible links to CNS-side effects.

## Conclusion

This is the first study measuring EFV metabolites in children. We observed distinct patterns of metabolite distribution, dependent on metabolizer phenotype based on composite CYP2B6 c.516G>T/CYP2B6 c.983T genotype, similar to what was described in adults. EFV phase II glucuronidated and sulfated hydroxy metabolites dominated in plasma for extensive and intermediate metabolizers. We also describe a recently identified substance, EFAdeg, hypothesized to be a degradation product of 8-OH-EFV. The combined 8-OH-EFV + EFAdeg/EFV ratio could differentiate between CYP2 B6 composite metabolizer phenotypes. This ratio increased over time among extensive metabolizers which was interpreted as a sign of autoinduction. CNS-related adverse effects were common but transient and mild and associated with supratherapeutic EFV levels and slow CYP2B6 metabolizer phenotype. We found no link between plasma levels of unconjugated/conjugated 7-OH-EFV, 8-OH-EFV or EFAdeg and CNS symptoms. Our findings highlight the complexity of EFV metabolism in children and underscore the need for further research to elucidate the relationship between EFV, EFV-metabolites and CNS adverse effects.

## Data Availability

The raw data supporting the conclusions of this article will be made available by the authors, without undue reservation.
