# Supplementary material for: Efavirenz metabolism and CNS toxicity in Ugandan children: impact of CYP2B6 genotype and plasma metabolite profiles
Source: Front Pharmacol. 2026 Apr 24;17:1778383. doi: 10.3389/fphar.2026.1778383 (PMC13153100; doi:10.3389/fphar.2026.1778383)
Supplement: Supplementary file 1 [file Supplementaryfile6.docx]

S.6. Predictors of plasma metabolite concentration and plasma metabolite/EFV ratios, investigated with multivariate restricted maximum likelihood regression (REML).

**S.6a. Predictors of log(e) 8-OH-EFV/EFV**

|  |  |  |  |  |  |  |
| --- | --- | --- | --- | --- | --- | --- |
| **log(e)8OH/EFV** | **Coefficient** | **Std. err.** | **z** | **P>z** | **[95% conf.** | **interval]** |
|  |  |  |  |  |  |  |
| Metabolizer phenotype | | |  |  |  |  |
| intermediate | 1.888897 | .1866851 | 10.12 | 0.000 | 1.523001 | 2.254793 |
| extensive | 2.294971 | .2083712 | 11.01 | 0.000 | 1.886571 | 2.703371 |
|  |  |  |  |  |  |  |
| Time on treatment (days) | -.0015137 | .0005312 | -2.85 | 0.004 | -.0025549 | -.0004725 |
| Age at treatment start (years) | .0442439 | .0281485 | 1.57 | 0.116 | -.0109261 | .099414 |
| Sex (female) | -.2356284 | .1351781 | -1.74 | 0.081 | -.5005725 | .0293158 |
| EFV dose (mg/kg) | -.0102038 | .0246165 | -0.41 | 0.679 | -.0584513 | .0380437 |
| Mean_Adherence (%) | .012163 | .016171 | 0.75 | 0.452 | -.0195316 | .0438576 |
| _cons | -4.878617 | 1.633181 | -2.99 | 0.003 | -8.079593 | -1.677642 |
|  |  |  |  |  |  |  |

**S.6b. Predictors of log(e) EFAdeg/EFV**

| **log(e)EFAdeg/EFV** | **Coefficient** | **Std. err.** | **z** | **P>z** | **[95% conf.** | **interval]** |
| --- | --- | --- | --- | --- | --- | --- |
|  |  |  |  |  |  |  |
| Time on treatment (days) | .0006636 | .0007148 | 0.93 | 0.353 | -.0007374 | .0020645 |
| Age at treatment start (years) | .0266082 | .026974 | 0.99 | 0.324 | -.0262599 | .0794764 |
| Sex (female) | -.2458691 | .1258846 | -1.95 | 0.051 | -.4925983 | .0008601 |
| EFV dose (mg/kg) | -.0203503 | .0274933 | -0.74 | 0.459 | -.0742362 | .0335357 |
| Mean_Adherence (%) | -.0103895 | .0151895 | -0.68 | 0.494 | -.0401604 | .0193813 |
|  |  |  |  |  |  |  |
| Metabolizer phenotype |  |  |  |  |  |  |
| intermediate | 1.532127 | .1792751 | 8.55 | 0.000 | 1.180754 | 1.8835 |
| extensive | 2.019021 | .198606 | 10.17 | 0.000 | 1.629761 | 2.408282 |
|  |  |  |  |  |  |  |
| _cons | -1.875601 | 1.543613 | -1.22 | 0.224 | -4.901027 | 1.149824 |

**S.6c. Predictors of log(e) 8-OH-EFV-tot/EFV**

| **log(e)8-OH-EFV-tot/EFV** | **Coefficient** | **Std. err.** | **z** | **P>z** | **[95% conf.** | **interval]** |
| --- | --- | --- | --- | --- | --- | --- |
|  |  |  |  |  |  |  |
| Metabolizer phenotype |  |  |  |  |  |  |
| intermediate | 2.025002 | .2365491 | 8.56 | 0.000 | 1.561374 | 2.488629 |
| extensive | 2.436072 | .2638917 | 9.23 | 0.000 | 1.918854 | 2.95329 |
|  |  |  |  |  |  |  |
| Time on treatment (days) | -.0003987 | .0003521 | -1.13 | 0.258 | -.0010887 | .0002914 |
| Age at treatment start (years) | .0446462 | .0328104 | 1.36 | 0.174 | -.0196609 | .1089534 |
| Sex (female) | -.478718 | .1697983 | -2.82 | 0.005 | -.8115166 | -.1459193 |
| EFV dose (mg/kg) | .0119174 | .0185237 | 0.64 | 0.520 | -.0243884 | .0482232 |
| Mean_Adherence (%) | .0121435 | .0185995 | 0.65 | 0.514 | -.0243108 | .0485978 |
| _cons | -1.897607 | 1.819899 | -1.04 | 0.297 | -5.464543 | 1.669328 |
|  |  |  |  |  |  |  |

**S.6d. Predictors of log(e) EFAdeg-tot/EFV**

| **log(e)EFAdeg-tot/EFV** | **Coefficient** | **Std. err.** | **z** | **P>z** | **[95% conf.** | **interval]** |
| --- | --- | --- | --- | --- | --- | --- |
|  |  |  |  |  |  |  |
| Metabolizer phenotype |  |  |  |  |  |  |
| intermediate | 1.816384 | .2252517 | 8.06 | 0.000 | 1.374899 | 2.25787 |
| extensive | 2.315648 | .2511442 | 9.22 | 0.000 | 1.823414 | 2.807881 |
|  |  |  |  |  |  |  |
| Time on treatment (days) | .0000648 | .000449 | 0.14 | 0.885 | -.0008153 | .0009448 |
| Age at treatment start (years) | .0495325 | .0321871 | 1.54 | 0.124 | -.0135531 | .112618 |
| Sex (female) | -.3243039 | .1619473 | -2.00 | 0.045 | -.6417148 | -.006893 |
| EFV dose (mg/kg) | -.0054522 | .0222137 | -0.25 | 0.806 | -.0489902 | .0380858 |
| Mean_Adherence (%) | .0003434 | .0183924 | 0.02 | 0.985 | -.035705 | .0363917 |
| _cons | -1.689967 | 1.824838 | -0.93 | 0.354 | -5.266585 | 1.88665 |

**S.6e. Predictors of log(e)(8-OH-EFV-tot+EFAdeg-tot)/EFV**

| **log(e)(EFAdeg-tot+8-OH-EFV-tot)/EFV** | **Coefficient** | **Std. err.** | **z** | **P>z** | **[95% conf.** | **interval]** |
| --- | --- | --- | --- | --- | --- | --- |
|  |  |  |  |  |  |  |
| Metabolizer phenotype |  |  |  |  |  |  |
| intermediate | 1.970738 | .2339576 | 8.42 | 0.000 | 1.51219 | 2.429287 |
| extensive | 2.40543 | .2609451 | 9.22 | 0.000 | 1.893987 | 2.916873 |
|  |  |  |  |  |  |  |
| Time on treatment (days) | -.000242 | .0003553 | -0.68 | 0.496 | -.0009383 | .0004544 |
| Age at treatment start (years) | .0483819 | .0325506 | 1.49 | 0.137 | -.0154161 | .1121798 |
| Sex (female) | -.4526569 | .1679201 | -2.70 | 0.007 | -.7817743 | -.1235396 |
| EFV dose (mg/kg) | .0133186 | .0188266 | 0.71 | 0.479 | -.0235808 | .050218 |
| Mean_Adherence (%) | .0129478 | .0183861 | 0.70 | 0.481 | -.0230883 | .048984 |
| _cons | -1.752626 | 1.801778 | -0.97 | 0.331 | -5.284045 | 1.778793 |

**S.6f. Predictors of log(e) 7-OH-EFV-tot mg/mL**

| Log(e) 7-OH-EFV-tot | Coefficient | Std. err. | z | P>z | [95% conf. | interval] |
| --- | --- | --- | --- | --- | --- | --- |
|  |  |  |  |  |  |  |
| Metabolizer phenotype |  |  |  |  |  |  |
| intermediate | -1.255675 | .1604929 | -7.82 | 0.000 | -1.570235 | -.9411148 |
| extensive | -1.544461 | .1850362 | -8.35 | 0.000 | -1.907126 | -1.181797 |
|  |  |  |  |  |  |  |
| Time on treatment (days) | .0006415 | .0004233 | 1.52 | 0.130 | -.0001882 | .0014713 |
| Age at treatment start (years) | .0290137 | .0236635 | 1.23 | 0.220 | -.0173659 | .0753934 |
| Sex (female) | .1485681 | .1196274 | 1.24 | 0.214 | -.0858972 | .3830334 |
| EFV dose (mg/kg) | .0231396 | .0192231 | 1.20 | 0.229 | -.0145369 | .0608161 |
| Mean_Adherence (%) | .0154845 | .0148873 | 1.04 | 0.298 | -.0136941 | .0446631 |
| _cons | 5.139735 | 1.481443 | 3.47 | 0.001 | 2.23616 | 8.043311 |

**S.6a-f.** Ninety-nine ART-naive Ugandan children aged 3-12 years initiated efavirenz (EFV)-based antiretroviral therapy and were classified into extensive, intermediate, or slow metabolizer phenotypes based on composite CYP2B6 516G>T/983T>C genotype, with 28, 54, and 15 children in each group, respectively. Mid-dose EFV and its metabolite plasma concentrations (ng/mL) were measured at 2, 6, 12, and 24 weeks. Predictors for log(e) metabolite/EFV ratios for 8-OH-EFV, 8-OH-EFV-tot, EFAdeg, EFAdeg-tot and for log(e)7-OH-EFV-tot (ng/mL) were investigated with REML. Random intercepts for individuals and random slope for treatment time were employed, while the remaining explaining variables were entered as fixed effects. Reference groups for categorical variables were “male” and “slow metabolizer phenotype”. The analyses included the following number of participants/observations:

6a: 92 participants with a total of 292 observations of 8-OH-EFV /EFV (in average 3,2 observations per individual).

6b: 86 participants with a total of 231 observations of EFAdeg/EFV in (in average 2,7 observations per individual).

6c: 94 participants with a total of 341 observations of 8-OH-EFV-tot/EFV (in average 3,6 observations per individual).

6d: 92 participants with a total of 323 observations of EFAdeg-tot/EFV in (in average 3,5 observations per individual).

6e: 94 participants with a total of 339 observations of 8-OH-EFV-tot+ EFAdeg-tot/EFV (in average 3,6 observations per individual).

6f: 89 participants with a total 322 observations of EFAdeg-tot/EFV in (in average 3,6 observations per individual).
