# Supplementary material for: Efavirenz metabolism and CNS toxicity in Ugandan children: impact of CYP2B6 genotype and plasma metabolite profiles
Source: Front Pharmacol. 2026 Apr 24;17:1778383. doi: 10.3389/fphar.2026.1778383 (PMC13153100; doi:10.3389/fphar.2026.1778383)
Supplement: Supplementary file 2 [file Supplementaryfile1.docx]

**GENEFA ADVERSE DRUG REACTION QUESTIONNAIRE**

Explain that the purpose of these questions is to help us find side effects that the patient may have experienced since he/she started the ARV treatment. At visit 1, explain to ART-naïve patients that we ask these questions *before and afte*r ART-start, to be able to differentiate already existing symptoms from drug related symptoms

If it is not known if the child did experience a particular symptom, please tick N/A.

Did you during the last 2 weeks experience any of the symptoms below?

1. **Dizziness…………………………………………… YES (1) [ ] NO (0) [ ] N/A[ ] Did it interfere with your daily activities (school, playing)?**

# NEVER (0) [ ] SOMETIMES (1) [ ] MOST TIMES (2) [ ] N/A [ ]

1. **Difficulty sleeping……………………………… YES (1) [ ] NO (0) [ ] N/A [ ]**

b) Did it interfere with your daily activities (school, playing)?

# NEVER (0) [ ] SOMETIMES (1) [ ] MOST TIMES (2) [ ] N/A[ ]

1. **Headache…………………………………………… YES (1) [ ] NO (0) [ ] N/A[ ]**

b) Did it interfere with your daily activities (school, playing)?

# NEVER (0) [ ] SOMETIMES (1) [ ] MOST TIMES (2) [ ] N/A[ ]

1. **Difficulty concentrating…………………………….YES (1) [ ] NO (0)[ ] N/A[ ]**

b) Did it interfere with your daily activities (school, playing)?

# NEVER (0) [ ] SOMETIMES (1) [ ] MOST TIMES (2) [ ] N/A[ ]

1. **Scary dreams ……………………………………. YES (1) [ ] NO(0) [ ] N/A[ ]**

b) Did it interfere with your daily activities (school, playing)?

# NEVER (0) [ ] SOMETIMES (1) [ ] MOST TIMES (2) [ ] N/A[ ]

6) Vivid dreams……………………………………..YES(1) [ ] NO(0)[ ] N/A[ ]

b) Did it interfere with your daily activities (school, playing)?

# NEVER (0) [ ] SOMETIMES (1) [ ] MOST TIMES (2) [ ] N/A[ ]

| **7) Confusion…………………………………………. YES (1) [ ]** | **NO (0) [ ]** | **N/A[ ]** |
| --- | --- | --- |
| **b) Did it interfere with your daily activities/playing? NEVER (0) [ ] SOMETIMES (1) [ ]** | **MOST TIMES (2) [ ]** | **N/A[ ]** |

1. **Did your feel sad/depressed (crying a lot, poor appetite, poor sleep) YES(1) [ ] NO(0) [ ] N/A[ ]**

b) Did it interfere with your daily activities (school, playing)

# NEVER (0) [ ] SOMETIMES (1) [ ] MOST TIMES (2) [ ] N/A[ ]

1. **Nausea …………………………………………….YES (1) [ ] NO(0) [ ] N/A[ ]**

b) Did it interfere with your daily activities (school, playing)?

# NEVER (0) [ ] SOMETIMES (1) [ ] MOST TIMES (2) [ ] N/A[ ]

1. **Vomiting…………………………………………..YES(1) [ ] NO(0) [ ] N/A[ ]**

b) Did it interfere with your daily activities (school, playing)?

# NEVER (0) [ ] SOMETIMES (1) [ ] MOST TIMES (2) [ ] N/A[ ]

1. **Abdominal pain…………………………………..YES (1) [ ] NO(0) [ ] N/A[ ]**

b) Did it interfere with your daily activities (school, playing)?

# NEVER (0) SOMETIMES (1) MOST TIMES (2) [ ] N/A[ ]

1. **Rash…………………………………………………YES (1) [ ] NO(0) [ ] N/A[ ]**

b) Did it interfere with your daily activities (school, playing)?

# NEVER (0) [ ] SOMETIMES (1) [ ] MOST TIMES (2) [ ] N/A[ ]

1. **Hallucinations (seeing things other people can't see) YES[ ] NO[ ] N/A[ ]**
2. **Suicidal tendencies requiring admission to hospital... YES[ ] NO[ ] N/A[ ]**
3. **Mental disturbances that required admission to hospital. YES[ ] NO [ ] N/A[ ]**
4. **Were you hospitalized due to any of the other symptoms?**

# NO[ ] YES[ ] N/A[ ]
