# Supplementary material for: Efavirenz metabolism and CNS toxicity in Ugandan children: impact of CYP2B6 genotype and plasma metabolite profiles
Source: Front Pharmacol. 2026 Apr 24;17:1778383. doi: 10.3389/fphar.2026.1778383 (PMC13153100; doi:10.3389/fphar.2026.1778383)
Supplement: Supplementary file 3 [file Supplementaryfile5.docx]

S5. Distribution of metabolite/efavirenz ratio, week 2-24 by composite CYP2B6 metabolizer phenotype

| Metabolite/EFV |  | Median  (IQR)  Min-Max | | | p-value | | | |
| --- | --- | --- | --- | --- | --- | --- | --- | --- |
|  | Week | EM | IM | SM | All | EM  vs IM | EM  vs SM | IM  vs SM |
| 8-OH-EFVtot/EFV | 2 | 4.45  (3.34-10.6)  1.14-72.89 | 2.73  (2.01-8.03)  0-32.97 | 0.71  (0.43-1.83)  0.22-2.28 | **0.0001** | **0.0234** | **0.0000** | **0.0000** |
|  | 6 | 5.29  (3.94-6.31)  3.2-17.47 | 3.76  (2.48-7.93)  0-39.94 | 0.49  (0.34-1.15)  0-2.07 | **0.0001** | **0.0167** | **0.0000** | **0.0000** |
|  | 12 | 5.72  (3.62-8.19)  1.16-36.23 | 4.4  (2.66-7.34)  0.35-38.55 | 0.51  (0.48-0.63)  0.3-7.47 | **0.0001** | 0.0745 | **0.0000** | **0.0000** |
|  | 24 | 5.49  (3.41-10.02)  1.43-40.58 | 3.5  (3.42-6.59)  0.13-15.94 | 0.61  (0.38-0.9)  0.18-2.19 | **0.0001** | **0.0077** | **0.0000** | **0.0000** |
| EFAdeg-tot/EFV | 2 | 1.52  (1.08-4.79)  0.52-21.42 | 0.96  (0.32-1.9)  0-4.74 | 0.27  (0.13-0.46)  0-0.65 | **0.0001** | **0.0011** | **0.0000** | **0.0005** |
|  | 6 | 1.59  (1.32-1.38)  0.98-6.03 | 0.96  (0.65-2.05)  0-9.68 | 0.22  (0.13-0.38)  0-0.91 | **0.0001** | **0.0006** | **0.0000** | **0.0000** |
|  | 12 | 1.42  (0.98-2.46)  0-13.02 | 1.32  (0.88–2.88)  0-12.71 | 0.16  (0.13-0.32)  0.06-1.73 | **0.0001** | 0.4026 | **0.0001** | **0.0000** |
|  | 24 | 1.71  (1.08-2.73)  0-7.43 | 1.2  (0.76-2.08)  0-4.96 | 0.19  (0.14-0.3)  0.06-1.13 | **0.0001** | **0.0169** | **0.0000** | **0.0000** |
| EFV_tot/EFV | 2 | 1.04  (1.02-1.06)  0.94-1.16 | 1.03  (1-1.06)  0.65-1.14 | 1.04  (1.01-1.06)  0.96-1.14 | 0.6592 | 0.5162 | 0.415 | 0.6563 |
|  | 6 | 1.05  (1.02-1.07)  0.96-1.11 | 1.04  (1.01-1.07)  0.95-1.04 | 1.02  (0.98-1.05)  0.94-1.09 | 0.1536 | 0.3043 | 0.0975 | 0.0939 |
|  | 12 | 1.03  (1-1.07)  0.9-1.16 | 1.03  (1-1.08)  0.96-1.15 | 1.01  (1-1.07)  0.94-1.13 | 0.5350 | 0.4540 | 0.3776 | 0.4137 |
|  | 24 | 1.05  (1.03-1.08)  0.98-1.13 | 1.05  (1.02-1.07)  0.95-1.11 | 1.04  (1-1.07)  0.97-1.08 | 0.6705 | 0.361 | 0.5699 | 0.4935 |
| EFAdeg /EFV | 2 | 0  (0-0.28)  0-3 | 0.08  (0-0.25)  0-0.48 | 0.03  (0-0.06)  0-0.11 | 0.4146 | 0.2491 | 0.4916 | 0.2657 |
|  | 6 | 0.24  (0.17-0.37)  0-0.86 | 0.1  (0-0.24)  0-0.82 | 0.05  (0-0.07)  0-0.23 | **0.0040** | **0.0033** | **0.0023** | 0.1351 |
|  | 12 | 0.27  (0.08-0.38)  0-1.15 | 0.14  (0-0.31)  0-0.93 | 0.02  (0-0.04)  0-0.13 | **0.0097** | 0.0513 | **0.0026** | **0.0249** |
|  | 24 | 0.34  (0.1-0.57)  0-1.01 | 0.19  (0.6-0.32)  0-1 | 0  (0.04-0.06)  0-0.18 | **0.0005** | 0.0250 | **0.0001** | **0.0024** |
| 8-OH-EFV /EFV | 2 | 0.18  (0.06-0.31)  0-1.74 | 0.15  (0.04-0.26)  0-0.68 | 0.03  (0.02-0.05)  0-0.07 | **0.0023** | 0.2411 | **0.0012** | **0.0013** |
|  | 6 | 0.19  (0.11-0.3)  0-0.56 | 0.13  (0.03-0.21)  0-0.71 | 0.02  (0.01-0.04)  0-0.1 | **0.0008** | 0.0674 | **0.0002** | **0.0014** |
|  | 12 | 0.19  (0.14-0.3)  0-0.99 | 0.13  (0.07-0.21)  0-0.74 | 0.02  (0-0.04)  0-0.11 | **0.0001** | **0.0091** | **0.0000** | **0.0001** |
|  | 24 | 0.16  (0.1-0.27)  0-0.69 | 0.06  (0.01-0.17)  0-0.7 | 0.02  (0.01-0.03)  0-0.05 | **0.0001** | 0.0355 | **0.0000** | **0.0001** |
| 7-OH-EFV-tot/EFV | 2 | 0.17  (0.11-0.23)  0-0.82 | 0.18  (0.1-0.24)  0-0.51 | 0.19  (0.09-0.31)  0.06-0.38 | 0.7701 | 0.4003 | 0.7219 | 0.5624 |
|  | 6 | 0.18  (0.11-0.26)  0-0.69 | 0.17  (0.09-0.26)  0-0.72 | 0.13  (0.09-0.25)  0-0.41 | 0.7343 | 0.3392 | 0.6560 | 0.5929 |
|  | 12 | 0.18  (0.11-0.26)  0-0.43 | 0.16  (0.11-0.24)  0-0.44 | 0.14  (0.08-0.23)  0.04-0.3 | 0.6258 | 0.2829 | 0.5113 | 0.5611 |
|  | 24 | 0.23  (0.11-0.32)  0-0.47 | 0.2  (0.08-0.31)  0-0.46 | 0.13  (0.01-0.28)  0.05-0.35 | 0.6524 | 0.4742 | 0.591 | 0.3607 |
| 7-OH-EFV/EFV | 2 | 0  (0)  0 | 0  (0)  0 | 0  (0)  0-0.02 | 0.9202 | 0.5000 | 0.0468 | 0.0398 |
|  | 6 | 0  (0-0)  0 | 0  (0-0)  0-0.03 | 0  (0-0)  0-0.01 | 0.9366 | 0.3075 | 0.2541 | 0.2530 |
|  | 12-24 | 0  (0-0)  0 | 0  (0-0)  0 | 0  (0-0)  0 | - | - | - | - |
| EFAdeg_tot + 8-OH-EFVtot/EFV | 2 | 5.77  (4.86 -15.39)  1.82-94.31 | 4.62  (2.26-10.59)  0-32.97 | 1.05  (0.56-2.22)  0.32-2.93 | **0.0001** | **0.0090** | **0.0000** | **0.0000** |
|  | 6 | 7.02  (4.97-8.69)  4.33-23.50 | 4.50  (3.17-8.99)  0-49.62 | 0.72  (0.46-1.40)  0-2.98 | **0.0001** | **0.0037** | **0.0000** | **0.0000** |
|  | 12 | 7.06  (5.25-10.41)  1.16-49.25) | 5.74  (3.25-10.10)  0.43-51.26 | 0.71  (0.62-0.92)  0.44-9.20 | **0.0001** | 0.1013 | **0.0000** | **0.0000** |
|  | 24 | 6.43  (4.98-12.98)  1.43-47.43 | 4.72  (3.33-9.20)  0.17-19.60 | 0.82  (0.52-1.22)  0.24-3.17 | **0.0001** | **0.0055** | **0.0000** | **0.0000** |
| EFAdeg+ 8-OH-EFV/EFV | 2 | 0.23  (0.06-0.64  0-4.74 | 0.28  0.08-0.54  0-1.06 | 0.06  0.03-0.10  0-0.18 | **0.0137** | 0.3454 | **0.0065** | **0.0094** |
|  | 6 | 0.45  (0.31-0.67)  0-1.09 | 0.26  0.05-0.47  0-0.99 | 0.05  0.02-0.11  0-0.28 | **0.0003** | **0.0069** | **0.000** | **0.0066** |
|  | 12 | 0.47  (0.22-0.65)  0-1.85 | 0.28  0.09-0.62  0-1.41 | 0.05  0.03-0.18  0-0.28 | **0.0001** | 0.0300 | **0.0000** | **0.0001** |
|  | 24 | 0.62  (0.27-0.76)  0-1.37 | 0.31  0.17-0.51  0-1.27 | 0.05  0.03-0.08  0-0.22 | **0.0001** | **0.0065** | **0.0000** | **0.0001** |

Ninety-nine ART-naive Ugandan children aged 3-12 years initiated efavirenz (EFV)-based antiretroviral therapy and were classified into extensive (EM), intermediate (IM), or slow metabolizer (SM) phenotypes based on their CYP2B6 516G>T/983T>C genotypes, with 28, 54, and 15 children in each group, respectively. Mid-dose EFV and its metabolite plasma concentrations (ng/mL) were measured at 2, 6, 12, and 24 weeks. Corresponding metabolite/EFV ratios were calculated based on plasma concentrations for unconjugated metabolites (7-OH-EFV/EFV, 8-OH-EFV/EFV, EFAdeg/EFV) and on plasma concentration for total concentrations of unconjugated + conjugated forms (EFV-tot/EFV, 7-OH-EFV-tot/EFV, and 8-OH-EFV-tot/EFV).Total concentrations included: **EFV-tot:** EFV + EFV-N-glucuronide; **7-OH-EFV-tot:** 7-OH-EFV + 7-OH-EFV-sulfate + 7-OH-EFV-glucuronide; **8-OH-EFV-tot:** 8-OH-EFV + 8-OH-EFV-sulfate + 8-OH-EFV-glucuronide. It was hypothesized that EFAdeg is a degradation product of 8-OH-EFV, existing in equilibrium with 8-OH-EFV. Therefore, the ratios of (EFAdeg + 8-OH-EFV)/EFV and (EFAdeg-tot + 8-OH-EFV-tot)/EFV are also presented. A Kruskal-Wallis test was used to detect significant differences in plasma concentration distributions, with p<0.05 considered significant. Further pairwise comparisons between EM, IM, and SM phenotypes were performed using the Conover test with Holm's correction for multiple testing, where p≤0.025 was considered significant. Statistically significant p-values are displayed in bold. Analyte concentrations below the lower limit of quantification were assigned a value of 0 in statistical analyses.
