# Supplementary material for: Efavirenz metabolism and CNS toxicity in Ugandan children: impact of CYP2B6 genotype and plasma metabolite profiles
Source: Front Pharmacol. 2026 Apr 24;17:1778383. doi: 10.3389/fphar.2026.1778383 (PMC13153100; doi:10.3389/fphar.2026.1778383)
Supplement: Supplementary file 4 [file Supplementaryfile3.docx]

S.3. Metabolite/efavirenz ratio week 2-24

| Week | Metabolite/EFV ratio | N | Min | Median | Interquartile  range | Max |
| --- | --- | --- | --- | --- | --- | --- |
| 2 | 8-OH-EFV-tot/EFV | 87 | 0,0 | 3,5 | (1,7-6,8) | 72,9 |
|  | 8-OH-EFV/EFV | 88 | 0,0 | 0,1 | (0,0-0,3) | 1,7 |
|  | 7-OH-EFV-tot/EFV | 94 | 0,0 | 0,2 | (0,1-0,3) | 0,8 |
|  | 7-OH-EFV /EFV | 94 | 0,0 | 0,0 | (0,0) | 0,0 |
|  | EFAdeg-tot/EFV | 86 | 0,0 | 0,9 | (0,3-1,7) | 21,4 |
|  | EFAdeg /EFV | 88 | 0,0 | 0,0 | (0,0-0,2) | 3,0 |
|  | EFV-tot/EFV | 94 | 0,6 | 1,0 | (1,0-1,1) | 1,2 |
|  | (EFAdeg-tot+8-OH-EFVtot) /EFV | 86 | 0,0 | 4,5 | (2,0-8,7) | 94,3 |
|  | (EFAdeg+8-OH-EFV) /EFV | 88 | 0,0 | 0,1 | (0,0-0,5) | 4,7 |
| 6 | 8-OH-EFV-tot/EFV | 88 | 0,0 | 3,7 | (2,2-6,2) | 39,9 |
|  | 8-OH-EFV/EFV | 88 | 0,0 | 0,1 | (0,0-0,3) | 0,6 |
|  | 7-OH-EFV-tot/EFV | 89 | 0,0 | 0,2 | (0,1-0,3) | 0,7 |
|  | 7-OH-EFV /EFV | 87 | 0,0 | 0,0 | (0,0) | 0,0 |
|  | EFAdeg-tot/EFV | 87 | 0,0 | 1,3 | (0,5-2,1) | 9,7 |
|  | EFAdeg /EFV | 90 | 0,9 | 0,1 | (0,0-0,3) | 0,9 |
|  | EFV-tot/EFV | 87 | 0,9 | 1,0 | (1,0-1,1) | 1,4 |
|  | (EFAdeg-tot+8-OH-EFV-tot) /EFV | 87 | 0,0 | 4,7 | (2,9-8,4) | 49,6 |
|  | (EFAdeg+8-OH-EFV) /EFV | 88 | 0,0 | 0,3 | 0,1-0,5 | 1,1 |
| 12 | 8-OH-EFV-tot/EFV | 82 | 0,3 | 4,1 | (2,4-7,1) | 38,6 |
|  | 8-OH-EFV/EFV | 82 | 0,0 | 0,1 | (0,0-0,2) | 1,0 |
|  | 7-OH-EFV-tot/EFV | 87 | 0,0 | 0,2 | (0,1-0,3) | 0,4 |
|  | 7-OH-EFV /EFV | 87 | 0,0 | 0,0 | (0,0) | 0,0 |
|  | EFAdeg-tot/EFV | 83 | 0,0 | 1,3 | (0,5-2,5) | 13,0 |
|  | EFAdeg /EFV | 83 | 0,0 | 0,1 | (0,0-0,3) | 1,2 |
|  | EFV-tot/EFV | 87 | 0,9 | 1,0 | (1,0-1,1) | 1,2 |
|  | (EFAdeg-tot+8-OH-EFVtot) /EFV | 82 | 0,4 | 5,4 | (3,0-9,8) | 51,3 |
|  | (EFAdeg+8-OH-EFV) /EFV | 82 | 0,0 | 0,3 | (0,1-0,6) | 1,8 |
| 24 | 8-OH-EFV-tot/EFV | 90 | 0,1 | 3,4 | (2,1-6,6) | 40,6 |
|  | 8-OH-EFV/EFV | 90 | 0,0 | 0,1 | (0,0-0,2) | 0,7 |
|  | 7-OH-EFV-tot/EFV | 92 | 0,0 | 0,2 | (0,1-0,3) | 0,5 |
|  | 7-OH-EFV/EFV | 92 | 0,0 | 0,0 | (0,0) | 0,0 |
|  | EFAdeg-tot/EFV | 90 | 0,0 | 1,2 | (0,7-2,3) | 7,4 |
|  | EFAdeg /EFV | 90 | 0,0 | 0,2 | (0,0-0,4) | 1,0 |
|  | EFV-tot/EFV | 92 | 0,9 | 1,0 | (1,0-1,1) | 1,1 |
|  | (EFAdeg-tot+8-OH-EFVtot) /EFV | 90 | 0,2 | 4,7 | (3,0-9,2) | 47,4 |
|  | (EFAdeg+8-OH-EFV) /EFV | 90 | 0,0 | 0,3 | (0,1-0,6) | 1,4 |

Ninety-nine ART-naive Ugandan children aged 3-12 years initiated efavirenz (EFV)-based antiretroviral therapy 2015-2016. Mid-dose EFV and metabolites plasma concentrations (ng/mL) were sampled at 2, 6, 12 and 24 weeks. N= number of successful measurements per analyte and visit. Analyte concentrations below the lower limit of quantification were assigned a value of 0 in statistical analyses. EFV and its phase I metabolites 7-OH-EFV and 8-OH-EFV were quantified both as unconjugated substance (EFV, 7-OH-EFV and 8-OH-EFV) and as total concentrations containing both unconjugated and conjugated substance (EFV-tot, 7-OH-EFV-tot and 8-OH-EFV-tot). The total concentrations included (EFV + EFV-N-glucuronide) for EFV-tot, (7-OH-EFV + 7-OH-EFV-sulfate + 7-OH-glucuronide) for 7-OH-EFV-tot and (8-OH-EFV + 8-OH-EFV-sulfate+ 8-OH-glucuronide) for 8-OH-EFVtot. EFAdeg was hypothesized to be an 8-OH-EFV degradation product in equilibrium with 8-OH-EFV, why the sums of EFAdeg + 8-OH-EFV and EFAdeg-tot+ 8-OH-EFV-tot are also displayed.
