# Supplementary material for: Efavirenz metabolism and CNS toxicity in Ugandan children: impact of CYP2B6 genotype and plasma metabolite profiles
Source: Front Pharmacol. 2026 Apr 24;17:1778383. doi: 10.3389/fphar.2026.1778383 (PMC13153100; doi:10.3389/fphar.2026.1778383)
Supplement: Supplementary file 5 [file Supplementaryfile2.docx]

S2. Efavirenz and metabolite concentrations (ng/mL), week 2-24

| Week | Metabolites | N | Min | Median | Interquartile  range | Max |
| --- | --- | --- | --- | --- | --- | --- |
| Week 0 | 8-OH-EFV-tot | 99 | 0 | 0 | 0 | 204 |
|  | EFAdeg-tot | 99 | 0 | 0 | 0 | 0 |
|  | EFV-tot | 99 | 0 | 0 | 0 | 0 |
|  | EFV | 99 | 0 | 0 | 0 | 0 |
|  | 7OH-EFV-tot | 99 | 0 | 0 | 0 | 0 |
|  | 8-OH-EFV | 99 | 0 | 0 | 0 | 0 |
|  | EFAdeg | 99 | 0 | 0 | 0 | 0 |
|  | 7-OH-EFV | 99 | 0 | 0 | 0 | 0 |
|  | EFAdeg_tot+8-OH-EFV-tot | 99 | 0 | 0 | 0 | 204 |
|  | EFAdeg_+ 8-OH-EFV | 99 | 0 | 0 | 0 | 0 |
| Week 2 | 8-OH-EFV-tot | 88 | 0 | 8874 | (4848-14586) | 43318 |
|  | EFAdeg-tot | 87 | 0 | 2334 | (1079-4323) | 10000 |
|  | EFV-tot | 95 | 0 | 2256 | (1435-4060) | 16126 |
|  | EFV | 95 | 0 | 2204 | (1358-3955) | 14951 |
|  | 7OH-EFV-tot | 95 | 0 | 421 | (229-807) | 5611 |
|  | 8-OH-EFV | 89 | 0 | 301 | (136-474) | 1953 |
|  | EFAdeg | 89 | 0 | 220 | (0-415) | 2836 |
|  | 7-OH-EFV | 95 | 0 | 0 | 0 | 296 |
|  | EFAdeg_tot+8-OH-EFV-tot | 87 | 0 | 11517 | (6010-21058) | 49240 |
|  | EFAdeg_+ 8-OH-EFV | 89 | 0 | 485 | (141-834) | 3717 |
| Week 6 | 8-OH-EFV-tot | 92 | 0 | 7703 | (5243-14878) | 40177 |
|  | EFAdeg-tot | 91 | 0 | 2673 | (1380-4209) | 9473 |
|  | EFV-tot | 94 | 0 | 2104 | (1285-3615) | 21982 |
|  | EFV | 94 | 0 | 2003 | (1285-3225) | 22836 |
|  | 7OH-EFV-tot | 93 | 0 | 363 | (165-737) | 3707 |
|  | 8-OH-EFV | 92 | 0 | 260 | (125-466) | 853 |
|  | EFAdeg | 92 | 0 | 244 | (0-514) | 3077 |
|  | 7-OH-EFV | 93 | 0 | 0 | 0 | 160 |
|  | EFAdeg_tot+8-OH-EFV-tot | 91 | 0 | 10461 | (7071-20083) | 47048 |
|  | EFAdeg_+ 8-OH-EFV | 92 | 0 | 538 | (182-934) | 3797 |
| Week 12 | 8-OH-EFV-tot | 87 | 0 | 9301 | (4976-16885) | 46087 |
|  | EFAdeg-tot | 88 | 0 | 2973 | (1408-6187) | 10000 |
|  | EFV-tot | 92 | 0 | 2570 | (1604-3814) | 20233 |
|  | EFV | 92 | 0 | 2473 | (1536-3762) | 18893 |
|  | 7OH-EFV-tot | 92 | 0 | 436 | (196-666) | 4355 |
|  | 8-OH-EFV | 87 | 0 | 351 | (169-507) | 1806 |
|  | EFAdeg | 88 | 0 | 344 | (0-582) | 3512 |
|  | 7-OH-EFV | 92 | 0 | 0 | 0 | 0 |
|  | EFAdeg_tot+8-OH-EFV-tot | 87 | 0 | 12147 | (6615-24292) | 53202 |
|  | EFAdeg_+ 8-OH-EFV | 87 | 0 | 709 | (297-1109) | 3994 |
| Week 24 | 8-OH-EFV-tot | 92 | 0 | 8916 | (5704-14729) | 44981 |
|  | EFAdeg-tot | 92 | 0 | 2716 | (1725-4648) | 10000 |
|  | EFV-tot | 94 | 0 | 2691 | (1549-4240) | 24364 |
|  | EFV | 94 | 0 | 2618 | (1524-3875) | 23715 |
|  | 7OH-EFV-tot | 94 | 0 | 447 | (237-863) | 5205 |
|  | 8-OH-EFV | 92 | 0 | 275 | (176-448) | 882 |
|  | EFAdeg | 92 | 0 | 467 | (78-817) | 2142 |
|  | 7-OH-EFV | 94 | 0 | 0 | 0 | 0 |
|  | EFAdeg-tot+8-OH-EFV-tot | 92 | 0 | 12179 | (7989-20220) | 51947 |
|  | EFAdeg + 8-OH-EFV | 92 | 0 | 842 | (346-1206) | 2539 |

Ninety-nine ART-naive Ugandan children aged 3-12 years were enrolled and initiated efavirenz (EFV)-based antiretroviral therapy 2015-2016. Mid-dose EFV and metabolites plasma concentrations (ng/mL) were sampled at 2, 6, 12 and 24 weeks. N= number of successful measurements per analyte and visit. For the statistical calculations, analyte plasma concentrations below the lower limit of quantification, were assigned a value of 0.

EFV and its phase I metabolites 7-OH-EFV and 8-OH-EFV were quantified both as unconjugated substance (EFV, 7-OH-EFV and 8-OH-EFV) and as total concentrations containing both unconjugated and conjugated substance (EFV-tot, 7-OH-EFV-tot and 8-OH-EFV-tot). The total concentrations included (EFV + EFV-N-glucuronide) for EFV-tot, (7-OH-EFV + 7-OH-EFV-sulfate + 7-OH-glucuronide) for 7-OH-EFV-tot and (8-OH-EFV + 8-OH-EFV-sulfate+ 8-OH-glucuronide) for 8-OH-EFVtot. EFAdeg was hypothesized to be an 8-OH-EFV degradation product in equilibrium with 8-OH-EFV, why the sums of EFAdeg + 8-OH-EFV and EFAdeg-tot+ 8-OH-EFV-tot are also displayed.
