# Supplementary material for: Efavirenz metabolism and CNS toxicity in Ugandan children: impact of CYP2B6 genotype and plasma metabolite profiles
Source: Front Pharmacol. 2026 Apr 24;17:1778383. doi: 10.3389/fphar.2026.1778383 (PMC13153100; doi:10.3389/fphar.2026.1778383)
Supplement: Supplementary file 6 [file Supplementaryfile4.docx]

S4. Distribution of plasma concentrations of efavirenz (EFV) and metabolites, week 2-24 by composite CYP2B6 metabolizer phenotype

| Plasma concentration  ng/ml | | Median  (IQR)  Min-Max | | | p-value | | | |
| --- | --- | --- | --- | --- | --- | --- | --- | --- |
|  | Week | EM | IM | SM | All | EM  vs IM | EM  vs SM | IM  vs SM |
| 8-OH-EFV-tot | 2 | 10632  (5620-15157)  0- 25871 | 8931  (4651-12854)  0-43318 | 5856  (4993-11873)  2398-29423 | 0.7024 | 0.5528 | 0.6341 | 0.3468 |
|  | 6 | 7622  (6649-9931)  0- 23028 | 9034  (5146-17226)  0-40177 | 7300  (3498-10641)  0-25268 | 0.5230 | 0.519 | 0.3163 | 0.4301 |
|  | 12 | 11747  (6745-17717)  0-27787 | 9657  (5087-18615)  0-46087 | 6698  (4119-9028)  1722-28148 | 0.2901 | 0.4709 | (0.1675 | 0.2019 |
|  | 24 | 9622  (6235-16884)  0- 42920 | 9524  (5768-14963)  0-44981 | 7153  (5071-9981)  2809-16777 | 0.2730 | 0.4635 | 0.1484 | 0.1937 |
| EFAdeg-tot | 2 | 3141  (1329-5835)  0-8491 | 1907  (922-3816)  0-10000 | 2354  (1222-4591)  0-9492 | 0.2850 | 0.1735 | 0.4487 | 0.3104 |
|  | 6 | 2594  (1918-3479)  0-7865 | 2539  (1147-4797)  0-9473 | 2919  (1391-4160)  0-7883 | 0.9865 | 0.4852 | 1.0000 | 0.8870 |
|  | 12 | 2841  (2061-4359)  0-9988 | 3184  (1411-7169)  0-10000 | 2096  (986-3531)  519-7797 | 0.3659 | 0.4155 | 0.2831 | 0.2848 |
|  | 24 | 3389  (1830-5079)  0-7244 | 2645  (1635-5988)  0-10000 | 2669  (2200-3229)  936-9225 | 0.7907 | 0.4894 | 0.5623 | 0.7648 |
| EFV-tot | 2 | 1686  (1217-2256)  0-7876 | 1976  (1384-3345)  103-12597 | 10689  (7300-12626)  5774-16126 | **0.0001*** | 0.0309 | **0.0000*** | **0.0000*** |
|  | 6 | 1602  (1090-1855)  0-2754 | 2282  (1244-3202)  0-1539 | 12087  (7285-15807)  109-21982 | **0.0001*** | **0.0016*** | **0.0000*** | **0.0000*** |
|  | 12 | 1944  (1547-2364)  0-4120 | 2540  (1541-3568)  0-17484 | 9896  (1596-16905)  3694-20233 | **0.0001*** | **0.0164*** | **0.0000*** | **0.0000*** |
|  | 24 | 1573  (1032-2261)  0-4140 | 2878  (1865-3505)  0-24364 | 13696  (8766-18660)  4280-22948 | **0.0001*** | **0.0000*** | **0.0000*** | **0.0000*** |
| EFV | 2 | 1577  (1181-2185)  0-7709 | 2120  (1358-3142)  111-12140 | 10052  (7078-12821)  5567-14951 | **0.0001*** | 0.0251 | **0.0000*** | **0.0000*** |
|  | 6 | 1525  (1073-1853)  0-2630 | 2237  (1197-2977)  0-15629 | 11621  (7155-14961)  111-22836 | **0.0001*** | **0.0014*** | **0.0000*** | **0.0000*** |
|  | 12 | 1909  (1486-2341)  0-4043 | 2456  (1425-3410)  0-17484 | 9864  (5121-16260)  3698-18893 | **0.0001*** | **0.0204*** | **0.0000*** | **0.0000*** |
|  | 24 | 1537  (1058-2196  0-3875 | 2786  (1767-3294)  0-23715 | 12725  (8192-18706)  3948-21324 | **0.0001*** | **0.0000*** | **0.0000*** | **0.0000*** |
| 7-OH-EFV-tot | 2 | 254  (154-491)  0-1161 | 401  (208-655)  0-1810 | 1542  (1087-2999)  441-5611 | **0.0001*** | 0.1446 | **0.0000*** | **0.0000*** |
|  | 6 | 221  (134-363)  0-1055 | 362  (181-633)  0-1849 | 1403  (868-2821)  0-3707 | **0.0001*** | **0.0214*** | **0.0000*** | **0.0000*** |
|  | 12 | 325  (168-502)  0- 619 | 381  (198-599)  0-3512 | 1420  (738-2233)  182-4355 | **0.0001*** | 0.1010 | **0.0000*** | **0.0000*** |
|  | 24 | 339  (170-442)  0-775 | 426  (205-839)  0-1506 | 1831  (1191-2394)  625-5205 | **0.0001*** | **0.0126*** | **0.0000*** | **0.0000*** |
| 8-OH-EFV | 2 | 279  (134-435)  0-1405 | 318  (135-521)  0-1953 | 268  (176-392)  0-996 | 0.8902 | 0.9536 | 0.8491 | 0.4258 |
|  | 6 | 207  (0-350)  0-853 | 283  (125-477)  0-770 | 204  (131-610)  0-843 | 0.7950 | 0.7896 | 0.6073 | 0.4815 |
|  | 12 | 364  (288-460)  0-1580 | 323  (126-516)  0-1806 | 284  (0-406)  0-1055 | 0.4265 | 0.4196 | 0.2979 | 0.2140 |
|  | 24 | 295  (142-498)  0-731 | 286  (183-452)  0-1736 | 199  (178-285)  0-1491 | 0.1682 | 0.4328 | 0.1173 | 0.0957 |
| EFAdeg | 2 | 0  (0-428)  0-2836 | 228  (0-411)  0-2239 | 342  (0-422)  0-1263 | 0.5513 | 0.2581 | 0.3822 | 0.4662 |
|  | 6 | 244  (0-490)  0-1199 | 225  (0-465)  0-2239 | 377  (0-1001)  0-3077 | 0.1558 | 0.2813 | 0.1819 | 0.0876 |
|  | 12 | 389  (203-624)  0-1694 | 298  (0-582)  0-3512 | 348  (0-403)  0-1262 | 0.6622 | 0.6092 | 0.4491 | 0.4306 |
|  | 24 | 593  (0-974)  0-2141 | 364  (202-766)  0-1736 | 620  (0-899)  0-1491 | 0.8104 | 0.7789 | 0.7338 | 0.4442 |
| 7-OH-EFV | 2 | 0  (0)  0 | 0  (0)  0 | 0  (0)  0-296 | 0.9200 | 0.5000 | 0.0441 | 0.0385 |
|  | 6 | 0  (0)  0 | 0  (0)  0 | 0  (0)  0-160 | 0.9139 | 0.500 | 0.0379 | 0.0319 |
|  | 12-24 | 0  (0)  0 | 0  (0)  0 | 0  (0)  0 | - | - | - | - |
| EFAdeg-tot + 8-OH-EFV-tot | 2 | 14549  (7126-21299)  0-33058 | 10189  (5492-15946)  0-49240 | 7583  (6818-13407)  3116-38915 | 0.6263 | 0.6893 | 0.3741 | 0.7367 |
|  | 6 | 10461  (8622-12884)  0-29665 | 11549  (6327-22640)  0-47048 | 10403  (6965-12807)  0-32613 | 0.7652 | 0.6893 | 0.3741 | 0.7367 |
|  | 12 | 14612  (9185-19205)  0-34563 | 12550  (6511-25553)  0-53202 | 8696  (6076-13961)  2493-34649 | 0.3397 | 0.4517 | 0.2343 | 0.2256 |
|  | 24 | 12674  (8180-21540)  0-50164 | 12964  (8138-21165)  0-51947 | 10070  (3771-13358)  6889-26002 | 0.3457 | 0.4791 | 0.1964 | 0.2515 |
| EFAdeg+ 8OH-EFV | 2 | 311  (134-888)  0- 3717 | 557  (135-816)  0-3017 | 665  (195-816)  0- 2259 | 0.6564 | 0.3453 | 0.5493 | 0.4990 |
|  | 6 | 604  (244-993)  0- 1786 | 501  (161-870)  0-2463 | 602  (204-816)  0- 3797 | 0.5944 | 0.4997 | 0.470 | 0.4702 |
|  | 12 | 754  (436- 933)  0-3226 | 695  (152--1141)  0-3993 | 618  (310-816)  0-1741 | 0.5069 | 0.4385 | 0.3872 | 0.2601 |
|  | 24 | 1048  (390-1325)  0- 2539 | 726  (407-1202)  0-2430 | 859  (178-816)  0- 1776 | 0.6977 | 0.5111 | 0.6585 | 0.3713 |

.

Ninety-nine ART-naive Ugandan children aged 3-12 years initiated EFV-based antiretroviral therapy. They were classified into extensive (EM), intermediate (IM), or slow metabolizer (SM) phenotypes based on their CYP2B6 516G>T/983T>C genotypes, with 28, 54, and 15 children in each group, respectively. Mid-dose EFV and its metabolite plasma concentrations (ng/mL) were measured at 2, 6, 12, and 24 weeks. Both unconjugated (EFV, 7-OH-EFV, and 8-OH-EFV) and total concentrations (unconjugated + conjugated forms: EFV-tot, 7-OH-EFV-tot, and 8-OH-EFV-tot) were quantified. Total concentrations included: **EFV-tot:** EFV + EFV-N-glucuronide; **7-OH-EFV-tot:** 7-OH-EFV + 7-OH-EFV-sulfate + 7-OH-EFV-glucuronide; **8-OH-EFV-tot:** 8-OH-EFV + 8-OH-EFV-sulfate + 8-OH-EFV-glucuronide. It was hypothesized that EFAdeg is a degradation product of 8-OH-EFV, existing in equilibrium with 8-OH-EFV. Therefore, the sums of EFAdeg + 8-OH-EFV and EFAdeg-tot + 8-OH-EFV-tot are also presented. Number of successful measurements per analyte and visit (weeks 2, 6, 12, and 24) were as follows:**8-OH-EFV-tot:** 88, 92, 87, 92, **EFV-tot:** 95, 91, 92, 94, **EFV:** 95, 94, 92, 94, **EFAdeg-tot:** 87, 91, 88, 92, **7-OH-EFV-tot:** 95, 93, 92, 94, **EFAdeg:** 87, 91, 88, 92, **8-OH-EFV:** 89, 92, 87, 92, **7-OH-EFV:** 95, 93, 92, 94. 7-OH-EFV was only quantifiable in two samples (two SM individuals) at week 2 and 6. A Kruskal-Wallis test was used to detect significant differences in plasma concentration distributions, with p<0.05 considered significant. Further pairwise comparisons between EM, IM, and SM phenotypes were performed using the Conover test with Holm's correction for multiple testing, where p≤0.025 was considered significant. Statistically significant p-values are displayed in bold.* Analyte concentrations below the lower limit of quantification were assigned a value of 0 in statistical analyses.
